# Hypoxia-Responsive Class III Peroxidases in Maize Roots: Soluble and Membrane-Bound Isoenzymes

**DOI:** 10.3390/ijms21228872

**Published:** 2020-11-23

**Authors:** Anne Hofmann, Stefanie Wienkoop, Sönke Harder, Fabian Bartlog, Sabine Lüthje

**Affiliations:** 1 Oxidative Stress and Plant Proteomics Group, Institute of Plant Science and Microbiology, Universität Hamburg, Ohnhorststrasse 18, 22609 Hamburg, Germany; anne.hofmann@uni-hamburg.de (A.H.); Fabian.Bartlog@studium.uni-hamburg.de (F.B.); 2 Department of Ecogenomics and Systems Biology, University of Vienna, Althanstrasse 14, 1090 Vienna, Austria; stefanie.wienkoop@univie.ac.at; 3 Center for Diagnostics Clinical Chemistry and Laboratory Medicine, Core Facility of Mass Spectrometric Proteomic, Campus Forschung N27, University Hospital Hamburg-Eppendorf (UKE), Martinistrasse 52, 20246 Hamburg, Germany; harder@uke.de

**Keywords:** aerenchyma, cell wall remodeling, class III peroxidases, hypoxia, maize roots, plasma membrane, respiratory burst oxidase homolog, *Zea mays* L.

## Abstract

Flooding induces low-oxygen environments (hypoxia or anoxia) that lead to energy disruption and an imbalance of reactive oxygen species (ROS) production and scavenging enzymes in plants. The influence of hypoxia on roots of hydroponically grown maize (*Zea mays* L.) plants was investigated. Gene expression (RNA Seq and RT-qPCR) and proteome (LC–MS/MS and 2D-PAGE) analyses were used to determine the alterations in soluble and membrane-bound class III peroxidases under hypoxia. Gel-free peroxidase analyses of plasma membrane-bound proteins showed an increased abundance of *Zm*Prx03, *Zm*Prx24, *Zm*Prx81, and *Zm*Pr85 in stressed samples. Furthermore, RT-qPCR analyses of the corresponding peroxidase genes revealed an increased expression. These peroxidases could be separated with 2D-PAGE and identified by mass spectrometry. An increased abundance of *Zm*Prx03 and *Zm*Prx85 was determined. Further peroxidases were identified in detergent-insoluble membranes. Co-regulation with a respiratory burst oxidase homolog (Rboh) and key enzymes of the phenylpropanoid pathway indicates a function of the peroxidases in membrane protection, aerenchyma formation, and cell wall remodeling under hypoxia. This hypothesis was supported by the following: (i) an elevated level of hydrogen peroxide and aerenchyma formation; (ii) an increased guaiacol peroxidase activity in membrane fractions of stressed samples, whereas a decrease was observed in soluble fractions; and (iii) alterations in lignified cells, cellulose, and suberin in root cross-sections.

## 1. Introduction

Plants worldwide have to cope with flooding events, and humanity has to manage the resulting agricultural yield loss of crop plants. The main reason for the dramatic effect of flooding seems to be the energy disruption, caused by the lack of oxygen needed in respiratory metabolisms. Fortunately, some plants can tolerate or adapt to this abiotic stress with the “low-oxygen escape strategy” or the “low-oxygen quiescence strategy” [1,2]. These adaptations to flooding-induced low-oxygen stress (hypoxia or anoxia) have been well studied so far [3,4,5]. Besides the energy disruption, the production of reactive oxygen species (ROS) and the imbalance of ROS-scavenging enzymes might be another factor for cell-damaging effects [6].

Reactive oxygen species (superoxide anion radical, hydroxyl radical, hydrogen peroxide, etc.) are produced even under physiological conditions via the aerobic pathway. They function as signaling molecules in plant growth, cell development, and programmed cell death [7,8]. Hypoxia has been shown to be responsible for ROS-induced oxidative stress [9,10]. The produced oxygen radicals cause lipid, nucleic acid, and protein oxidation, as well as total cell damage [6]. Increased ROS levels can be reduced by ROS-scavenging molecules (ascorbate and glutathione) or enzymes (superoxide dismutase, catalase, ascorbate peroxidases, glutathione peroxidases, and class III peroxidases).

Maize (*Zea mays* L.) belongs to the waterlogging-tolerant plant species that adapt to hypoxia by developing aerenchyma in roots for ventilation. It has been demonstrated that hypoxia-induced development of aerenchyma occurred in maize roots after 12 to 60 h [11]. Reactive oxygen species and cell wall degradation play a crucial role in the formation of aerenchyma by several stressors. A function of respiratory burst oxidase homologs (Rboh) in programmed cell death during aerenchyma formation has been suggested by a strong upregulation of those genes [12,13,14]. Additionally, some wetland plant species form a suberin barrier at the outer cell layers of roots to reduce radial oxygen loss (ROL) from aerenchyma [15]. Accumulation of suberin at the hypodermal/exodermal cell layers and deposition of lignin was observed in adventitious roots under waterlogged soil conditions [16]. It was concluded that ROL barrier formation contributes to higher waterlogging tolerance in plants. Class III peroxidases were upregulated by hypoxia [11,14]. Although guaiacol peroxidase activity of plant extracts has been used as a general stress marker for a long time [17], results might be not clear, because of the high amount of isoenzymes that could be differentially regulated. To distinguish between several isoenzymes and to identify low-abundant peroxidases involved in a specific stress response, proteomic approaches are state of the art [18,19]. Although peroxidases involved in biotic and abiotic stress are well studied, the impact of hypoxia, e.g., induced by flooding, on class III peroxidases is still rarely investigated [12,20,21].

The class III peroxidases are heme-containing proteins of the secretory pathway in higher plants with a high number of isoforms. The secretory pathway delivered the glycosylated peroxidases into the apoplast and cell wall, but also to the vacuole, plasma membrane (PM), and thylakoid [22,23]. Due to their peroxidative or hydroxylic reaction cycles, peroxidases are involved in cell wall-related reactions, metabolic pathways, and stress-related processes [23,24,25]. Besides the ROS scavenging, peroxidases take part in the final steps of lignin and suberin synthesis [26,27]. Sinapyl and cinnamyl alcohols are precursors of lignin monomers [28], whereas ferulic, caffeic, sinapinic, and *p*-coumaric acid are precursors of suberin [27]. Peroxidases mediate the crosslinking of cell wall compounds in response to different stimuli [29]. In grasses, arabinose and arabinoxylans are crosslinking by peroxidase-generated diferulates [30,31]. So far, 158 peroxidases have been identified in the maize (*Zea mays* L.) genome (RedoxiBase, as of 6 October 2020). A majority of maize peroxidase genes appear expressed in root tissues [32,33]. However, evidence has been presented for localization of about 25% of the class III peroxidases in the PM [23]. To date, four peroxidases have been purified from PM of maize roots and were characterized biochemically [34,35]. Evidence for a function of these peroxidases in biotic and abiotic stress has been given by a proteomic approach [19].

In the present study, we used a systems biological approach to identify hypoxia-responsive class III peroxidases with a special focus on PM-bound isoenzymes. We also discuss their possible functions in maize roots.

## 2. Results

### 2.1. RNA Sequence Analyses

**Figure 1:**
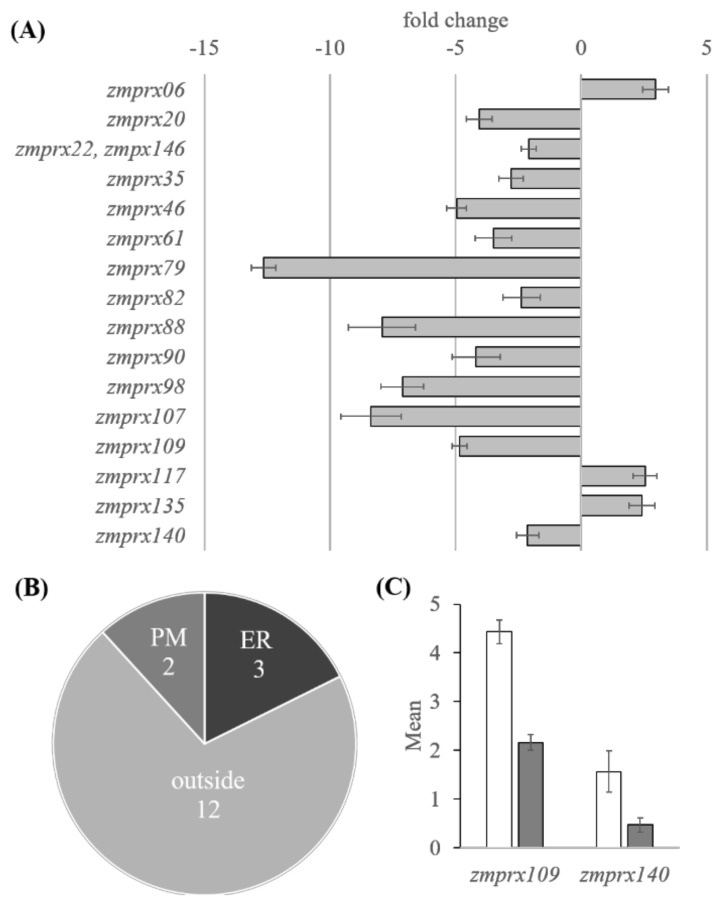
Differential regulation and putative localization of class III peroxidases under hypoxia. From three non-stressed and 24 h hypoxia-stressed root samples, expression profiles were calculated as Fragments Per Kilobase Million (FPKMs). Differentially expressed gene (DEG) results were performed on a comparison pair (stressed versus controls), using FPKM with a fold change >2 and Student’s *t*-test *p* < 0.05. (**A**) Differential expressed peroxidases (excluding pseudogenes) with their fold change expression and (**B**) their predicted localization, using PSORT (http://psort1.hgc.jp/form.html) at either the plasma membrane (PM), the endoplasmic reticulum (ER) or “outside” (apoplast, cell wall). (**C**) Gene expression (mean = mean of normalized signal for each sample or group) of the two putative plasma membrane-bound class III peroxidases that were differentially expressed in controls (white columns) and hypoxia-stressed samples (gray columns). Error bars indicate standard deviation (three biological replicates).

In the RNA Sequence (RNA Seq) analyses of control and stressed maize root samples, 152 Prx transcripts (135 genes) and 20 Rboh transcripts (11 genes) were identified. Pseudogenes were excluded from the analyses. Some of the peroxidases, catalogued by RedoxiBase database, could not be detected. This contains z*mprx12*, *zmprx138*, *zmprx36*, *zmprx40*, *zmprx41*, *zmprx66*, *zmprx75*, *zmprx 01_W64A*, *zmprx100_35A19*, *zmprx108_35A19*, *zmprx03_Du101*, *zmprx03_F66*, *zmprx03_Lan496*, *zmprx03_W64A*, and *zmprx03_Wis93*. The statistical analysis of the RNA Seq data revealed a significant upregulation of *zmrboh10* and 17 differentially expressed class III peroxidase genes (DEGs), of which three were upregulated and 13 were downregulated based on 2-fold change and *p*-value of *p* < 0.05 of comparison pair (hypoxia versus control) (Figure 1A and Supplementary Materials Table S1A,B). In silico prediction of the 17 class III peroxidases showed localization in the endoplasmic reticulum (*zmprx35*, *zmprx46*, and *zmprx135*), the PM (*zmprx109* and *zmprx140*), and “outside”, i.e., apoplast or cell wall (Figure 1B). The two PM predicted peroxidases, *zmprx109* and *zmprx140*, were 2–5-fold downregulated in stressed maize plants (*zmprx109* with mean of 2.16 ± 0.16 and *zmprx140* with mean of 0.47 ± 0.15) compared to controls (*zmprx109* with mean of 4.44 ± 0.24 and *zmprx140* with mean of 1.56 ± 0.43) (Figure 1A,C).

Besides these peroxidases, genes of key enzymes of cell wall synthesis, degradation, and reinforcement were found to be differentially regulated by hypoxia (Supplementary Materials Table S1C). The phenylpropanoid pathway was presented by caffeoylshikimate esterase (*zmcse*, A0A1D6N7M7) and several cinnamyl alcohol dehydrogenases (*zmcad*). The expression level of *zmcse* increased 17-fold, whereas that of *zmcad1* (B4FAJ0) was moderately upregulated. Expression levels of *zmcad6* (B4FR97) and *zmcad* (O24562) decreased significantly, 2.5-fold and 2.9-fold, respectively. Most of the *zmcad* genes were downregulated. The expression level of cellulose synthase 5 (*zmcesa*, A0A1D6L8J3) increased 2.8-fold, whereas two other *zmcesa* genes (B4FJI1 and B6TTA1) were significantly downregulated. The expression of further *zmcesa* genes decreased. Omega-hydroxypalmitate O-feruloyl transferase (*zmhht*, B4FV3), a key enzyme of suberin biosynthesis, showed a 2.9-fold downregulation. For cell wall degradation, two expansins (*zmexpl3*, B4FL59, and *zmexp3A*, A0A1D6EVK8) were significantly upregulated by hypoxia, whereas 20 other expansins were downregulated. Finally, caffeoyl-CoA *O*-methyltransferase 1 (*zmccoaomt*, B6UF45), involved in cell wall reinforcement, was downregulated.

Among the 15 dirigent (*zmdir*) genes, one transcript (B6U4X5) was 2.1-fold upregulated, six were downregulated and eight were not differentially regulated by hypoxia (Supplementary Materials Table S1C). Expression levels of eighteen fasciclin-like arabinogalactan transcripts (*zmfla*) were either downregulated (*zmfla2*, B6SZA0; *zmfla7*, B6SHU5; *zmfla10*, A0A096SZF8; *zmfla7*, B4FB81; *zmfla6*, B4F7Z4) or not significantly affected. A glycerophosphodiesterase (*zmgdpd3*, A0A1D6KIK2) was 2.7-fold upregulated.

### 2.2. Hydrogen Peroxide Determination, Total Guaiacol Peroxidase Activity and Abundance

**Figure 2:**
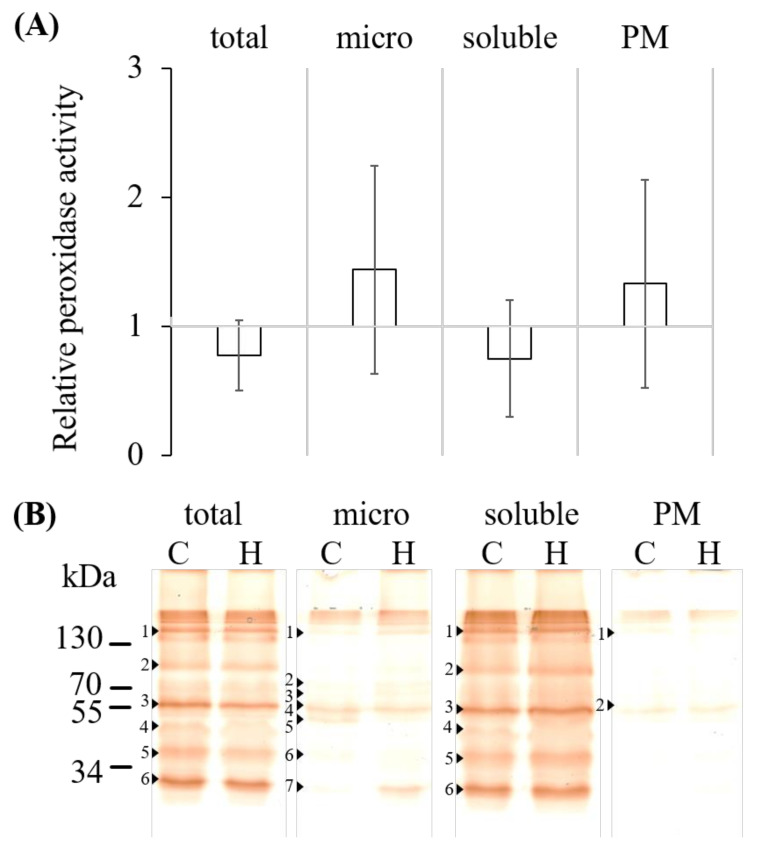
Total guaiacol peroxidase activity and abundance of control and hypoxia-stressed maize roots. Non-stressed (C) and 24 h hypoxia-stressed root samples (H) were divided into total extracts (total) and the sub-proteomes soluble proteins, microsomes (micro), and plasma membranes (PM). (**A**) Using the substrate guaiacol, the total activity was detected spectrophotometrically of the cellular fractions. Data of the stressed samples were related to the controls (three biological and three technical replicates). (**B**) The abundances of guaiacol peroxidases were detected after separation by 11% polyacrylamide gels of the cellular fractions. Peroxidase bands (nos. 1-7) were marked by arrows. Shown is one representative replicate.

Control and 24 h hypoxia-stressed root samples were divided into total extracts and the sub-proteomes soluble proteins, microsomes, and PM. The PM fraction showed an enrichment of the H^+^-ATPase and a lower amount of V-PPase and Cox2 signals compared to the corresponding microsomal proteins (Supplementary Materials Figure S1). Total extracts of 24 h hypoxia-stressed root tissue showed a significant (*p* < 0.01) 1.5-fold increased level of hydrogen peroxide compared to controls. The quantification of hydrogen peroxide in those samples revealed amounts of 227 ± 12 µM in control and 365 ± 19 µM in stressed samples. The guaiacol peroxidase activities decreased by about 25% in total fractions of stressed samples (5.95 ± 4.73 µmol min^−1^ mg^−1^) compared to controls (7.82 ± 5.51 µmol min^−1^ mg^−1^) as well as by about 30% in soluble fractions of stressed samples (30.68 ± 23.39 µmol min^−1^ mg^−1^) compared to controls (45.55 ± 25.04 µmol min^−1^ mg^−1^). Contrary to this, membrane fractions showed a about 33% increased activity of 0.97 ± 1.04 µmol min^−1^ mg^−1^ (stressed) to 0.64 ± 0.45 µmol min^−1^ mg^−1^ (controls) in microsomes as well as an about 44% increase of 0.71 ± 0.46 µmol min^−1^ mg^−1^ (stressed) to 0.54 ± 0.23 µmol min^−1^ mg^−1^ (controls) in PM (Figure 2A). Separation of those fractions by modified SDS-PAGE revealed a higher abundance of class III peroxidases in total and soluble fractions compared to membrane fractions (Figure 2B). Several peroxidase isoforms were detected with different protein masses or complexes of proteins. Six bands (nos. 1–6) in total and soluble fraction (148, 103, 70, 55, 43, and 31 kDa) could be determined that showed only a slight increase in abundance in stressed samples compared to controls. Seven bands (nos. 1–7) in microsomal fractions (147, 84, 77, 65, 59, 41, and 29 kDa) were visible, of which the 84, 77, and 29 kDa bands showed an increased abundance of more than 2.0-fold contrary to the 59 kDa band that showed a decrease of 0.4-fold compared to controls. Only two bands (nos. 1 and 2) in PM fractions (148 and 65 kDa) could be seen with no significant difference in abundance between control and stressed samples.

### 2.3. Gel-Free Peroxidase Analyses

**Figure 3:**
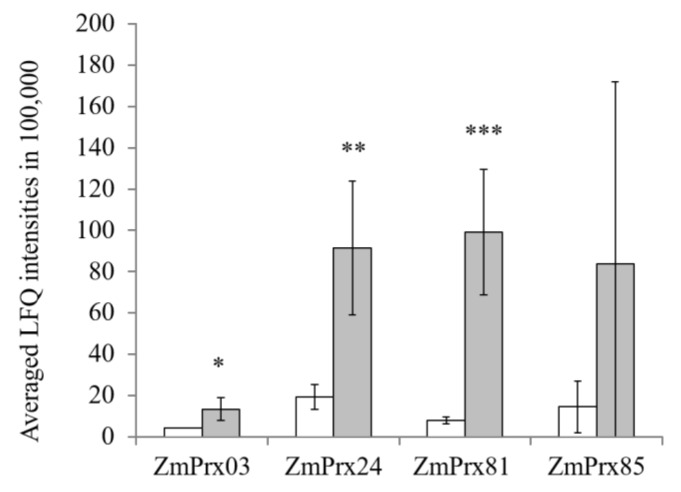
Mass spectrometry analyses of the plasma membrane for class III peroxidases in control and hypoxia-stressed maize roots. Plasma membranes of controls (white columns) and 24 h hypoxia-stressed maize roots (gray columns) were used for mass spectrometry analyses. Label-free quantifications (LFQ) data were determined with MaxQuant software and used for further statistical analyses. Error bars indicate standard deviation. Asterisks indicate significances (*p* < 0.05 *, *p* < 0.01 **, and *p* < 0.001 ***) determined with Student’s *t*-test for three biological and two technical replicates per treatment.

The gel-free approach revealed four class III peroxidases in PM fractions of control and stressed roots. Here, *Zm*Prx03 (A0A1D6LYW3, two unique peptides), *Zm*Prx24 (B4FHG3, five unique peptides), *Zm*Prx81 (B4FG39, seven unique peptides), and *Zm*Prx85 (A0A1D6E530, three unique peptides) were identified (Supplementary Materials Table S2A). Abundance of *Zm*Prx03, *Zm*Prx81 and *Zm*Prx24 significantly increased in stressed PM samples, whereas *Zm*Prx85 abundance shows a non-significant tendency to increase (Figure 3).

Besides these peroxidases, increased abundances were found for several cell wall-related proteins (Supplementary Materials Table S2B). Among these were two dirigent proteins (B4FV87 and B6T6D2), FLA10 (C0PD01), and two glycerophosphodiester phosphodiesterases (GDPDL3, A0A1D6HBU2, and C0PGU8) that showed a weak increase in hypoxia-stressed samples.

### 2.4. Gel-Based Peroxidase Analyses

**Figure 4:**
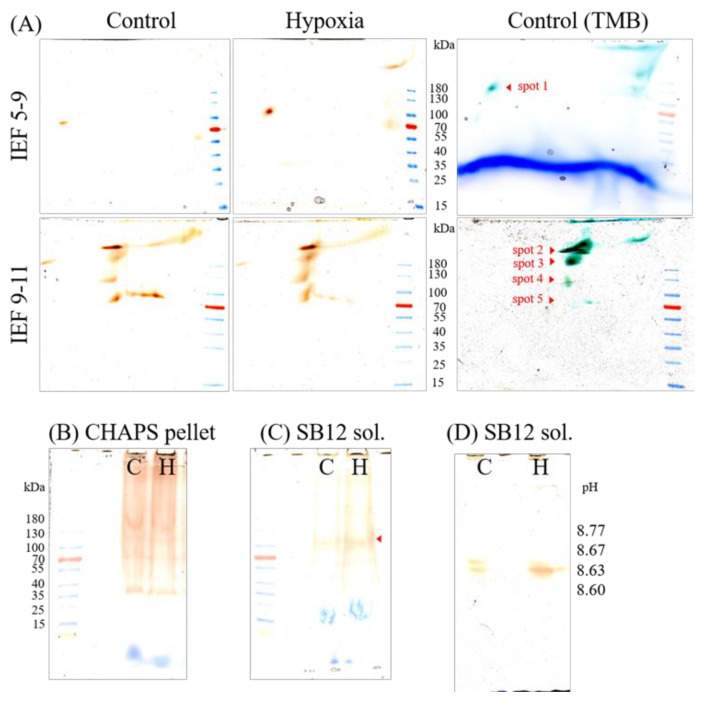
Abundance of guaiacol peroxidases under hypoxia-stress. Plasma membranes (250 µg total protein) of control (C) and 24 h hypoxia-stressed (H) maize roots were solubilized with 8% CHAPS for 1 h. The supernatant was separated on (**A**) native IEF gels pH 5–8 and pH 9–11 followed by 4–18% non-denaturing polyacrylamide gels in second dimensions. The remaining pellet was either separated on 4–18% non-denaturing gels (**B**) or solubilized with SB12 (protein-detergent ratio of 1:7) for 1 h. The SB12 supernatant (sol.) was loaded on 4–18% non-denaturing gels (**C**) and IEF gels pH 9–11 (**D**). Class III peroxidases were visualized by staining with hydrogen peroxide and guaiacol (orange color). No or only weak signal could be determined in the pellet after SB12 solubilization (data not shown). For MS analyses, gels were stained with TMB (blue color). The protein spots and bands, indicated with red arrows, were identified by mass spectrometry. Molecular weight in kDa was determined using a protein standard (PageRuler Prestained Protein Ladder, Thermo Scientific, Waltham, MA, USA). For further details, see the text. SB12, n-dodecyl-*N*, *N*-dimethyl-3-ammonio1-propanesulfonate; CHAPS, 3-[(3-Cholamidopropyl)dimethylammonio]1-propanesulfonate; TMB, 3,3′,5,5′-Tetramethylbenzidine.

Plasma membrane that was solubilized with 3-[(3-Cholamidopropyl)dimethylammonio]-1-propanesulfonate (CHAPS) and separated on native isoelectric focusing (IEF) gels with a pH range from 5 to 9 revealed one recurring spot at acidic pH (Figure 4A). This spot was identified by MS as *Zm*Prx85 with a coverage of 27%. The isoelectric point (pI) and molecular weight (MW in kDa) of this peroxidase (spot no. 1) ranged from pI 4.73 ± 0.11 to 109 ± 11 kDa in control samples and not significantly different from pI 4.82 ± 0.14 to 106 ± 11 kDa in stressed samples. The analysis of the spot intensity revealed a 2.2-fold increased abundance of *Zm*Prx85 in stressed samples compared to controls (four biological and two technical replicates; Figures S3 and S4). Besides, four main spots at pH > 9 were determined in these gels but were furthermore separated on IEF gels with pH range from 9 to 11 (Figure 4A). These spots were identified by MS as *Zm*Prx101 (B4FU88) with pI 8.8 ± 0.1 and 290 ± 37 kDa (spot no. 2), as *Zm*Prx03 with pI 8.7 ± 0.5 and 227 ± 27 kDa (spot no. 3), as *Zm*Prx01 (A5H8G4) and *Zm*Prx03 with pI 8.6 ± 0.4 and 131 ± 9 kDa (spot no. 4), and as *Zm*Prx01 with pI 8.7 ± 0.1 and 82 ± 5 kDa (spot no. 5), respectively. The pI and MW of these spots did not differ significantly between control and stressed samples. The analyses of the abundances showed an increase of *Zm*Prx101 (spot no. 2, 1.3-fold), *Zm*Prx03 (spot no. 3, 2.3-fold), *Zm*Prx01 and *Zm*Prx03 (spot no 4, 3.6-fold), and *Zm*Prx01 (spot no. 5, 1.2-fold) of the stressed samples compared to the control samples, respectively. Spot no. 4 contains the two class III peroxidases *Zm*Prx01 and *Zm*Prx03. In the spot of the control sample, *Zm*Prx01 was the dominant peroxidase (with six peptides) compared to the spot of the stressed sample (with three peptides). Contrary to this, *Zm*Prx03 was the dominant peroxidase in the spot of the stressed sample (with six peptides) compared to the spot of the control sample (with three peptides). Besides these five representative spots, that were found in all biological and technical replicates, some additional spots (spots nos. 6–8) appeared in only some biological samples (Supplementary Materials Figure S2). Four peroxidases (*Zm*Prx24, *Zm*Prx87 (B4FSW5), *Zm*Prx118 (B4FK72), and *Zm*Prx85) were identified in spot no. 6 with pI 8.03–8.3 and 31 ± 2 kDa. Spot no. 7 contained *Zm*Prx85 with pI 8.0–8.4 and 123 ± 9 kDa, and spot no. 8 revealed *Zm*Prx01 with pI 8.7–8.8 and 91 ± 12 kDa. The abundance of these spots was higher in stressed samples compared to control samples (Supplementary Materials Figure S2). Co-separation of these peroxidases with *Zm*RbohB (A0A1D6MT17) and/or *Zm*Rboh04 (A0A1D6QI90) was found in spots nos. 2–5 of the 2D-PAGE (Supplementary Materials Table S3).

Class III peroxidases were detected in the remaining PM pellets of controls and stressed samples (Figure 4B). After solubilization of these pellets with n-dodecyl-*N*,*N*-dimethyl-3-ammonio-1-propanesulfonate (SB12), *Zm*Prx81 (B4FG39) and *Zm*Prx85 were found in controls and, additionally, *Zm*Prx01 and *Zm*Prx70 (A5H452) were found in stressed samples (Figure 4C). In IEF gels with pH 9–11, the SB12 solubilized supernatant showed guaiacol positive proteins at pH 8.6 (Figure 4D). All identified class III peroxidases with the corresponding peptides can be found in Supplementary Materials Table S3.

### 2.5. RT-qPCR Analyses

**Figure 5:**
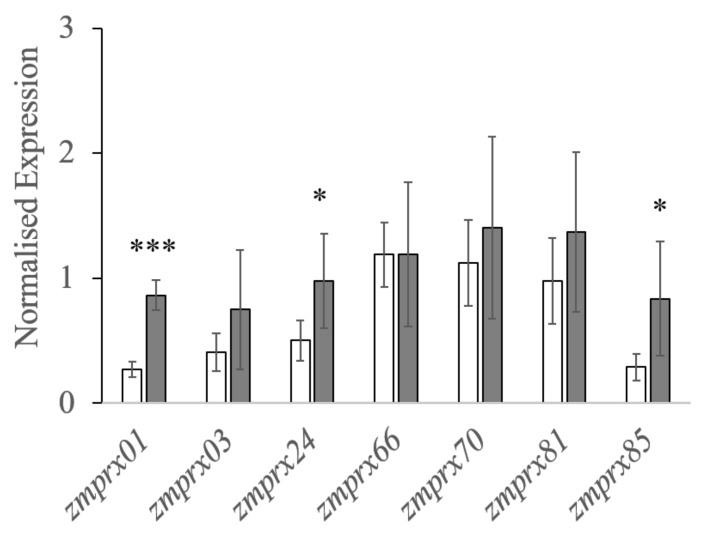
Expression profiles of class III peroxidases identified in the plasma membrane of maize roots. Total RNAs of non-stressed (white columns) and 24 h hypoxia-stressed roots (gray columns) were extracted for gene expression analyses (RT-qPCR) with SYBRGreen. Expression was normalized to *zmtufM* as housekeeping gene. Significancies, calculated with Student´s *t*-test, are marked with (*p* < 0.05 * and *p* < 0.001 ***) for three biological and two technical replicates per treatment.

The regulation of the six PM-bound class III peroxidases, identified by gel-free and gel-based analyses, was investigated by real-time quantitative polymerase chain reaction (RT-qPCR) (Figure 5). Although all genes were upregulated, a significant increase was found for *zmprx01* (3.2-fold), *zmprx24* (1.9-fold), and *zmprx85* (2.9-fold), whereas expression of *zmprx03* (1.8-fold), *zmprx70* (1.3-fold), and *zmprx81* (1.4-fold) showed higher levels compared to controls. Additionally, the expression of *zmprx66* was not increased in comparison to controls. The housekeeping gene, used in RT-qPCR (*zmtufM*, Q9FUZ6), could not be found in the RNA Seq data. The NCBI blast with the corresponding sequence led to the protein (NP_001141314) with less than 100% coverage. This protein had a slight non-significant decrease in expression (−1.4-fold change) in RNA Seq analyses, as well as another elongation factor (gene entry 542581, −1.5-fold change).

### 2.6. Root Cross-Sections and In Vivo Root Staining

**Figure 6:**
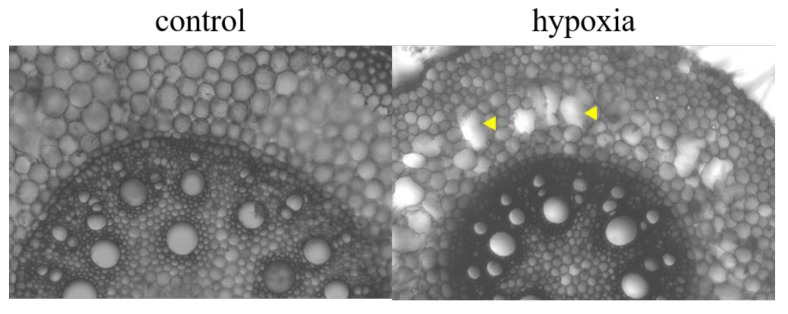
Aerenchyma formation in the mature zone of maize primary root cross-sections. Control and 24 h hypoxia-stressed maize roots were cross-sectioned, by hand, for observation of aerenchyma (indicated with arrows). Images taken with 10x magnification using a Leica DM500 binocular microscope.

During cross-sectioning, aerenchyma formation in the root cortex was visible in some but not all of the stressed plants, while there were no such signs in control plants (Figure 6).

**Figure 7:**
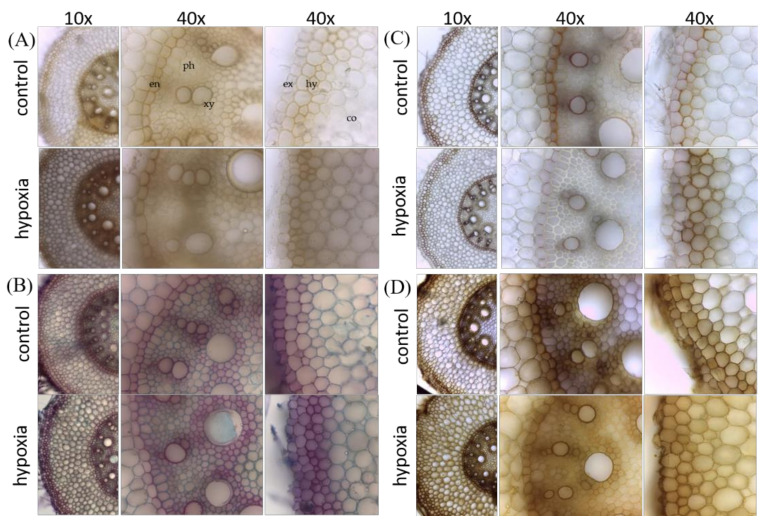
Visualization of lignin, its precursors, and cellulose in the mature zone of primary root cross-sections from control and hypoxia-stressed maize plants. Control and 24 h hypoxia-stressed maize roots were cross-sectioned by hand and analyzed with (**A**) Mäule staining for syringyl-rich polyphenols (deep-red color), (**B**) fuchsin, chrysoidin, and Astra blue (FCA) staining for lignified cells (red color) and non-ligneous cells (blue color), (**C**) Phloroglucinol staining for lignified cells (pink color), and (**D**) chlorine–zinc–iodine staining for cellulose (violet color). For detailed explanation of the colours, see methods. Co, cortex; en, endodermis; ex, exodermis; hy, hypodermis; ph, phloem; xy, xylem.

Mäule staining revealed overall darker staining of stressed samples compared to controls including hypodermis, cortical cell region, and vascular sclerenchyma cells (Figure 7A). The yellow-brownish color indicates the presence of guaiacyl lignin monomers in both, controls and stressed samples. No red staining was observed that would show the presence of syringyl lignin monomers. Etzold staining with fuchsin, chrysoidin, and Astra blue (FCA) revealed overall stronger staining of stressed samples compared to controls (Figure 7B). Phloem and pith of the vascular sclerenchyma cells seem non-lignified (blue appearance), hypodermis, endodermis, and xylem cells are lignified (pink appearance). In stressed cells, more hypodermic layers are lignified compared to controls. Phloroglucinol revealed strong staining of hypodermis, endodermis, and xylem vessels. All of those cells were less stained in stressed samples compared to controls (Figure 7C). Controls show strong yellowish staining of the Casparian band in endodermal cell walls. The exodermis and xylem vessels of controls have a positive pink color, indicating lignified cells, which is not observed in stressed samples. There are more yellow-brownish stained hypodermic cell layers in stressed samples compared to controls. With chlorine–zinc–iodine staining, a positive cellulose reaction in cortical and vascular sclerenchyma cells (phloem and pith) of controls was observed, that did not appear in stressed samples. Hypodermis, endodermis, and xylem cells are stained in a brownish color with less intensity in stressed samples (Figure 7D).

**Figure 8:**
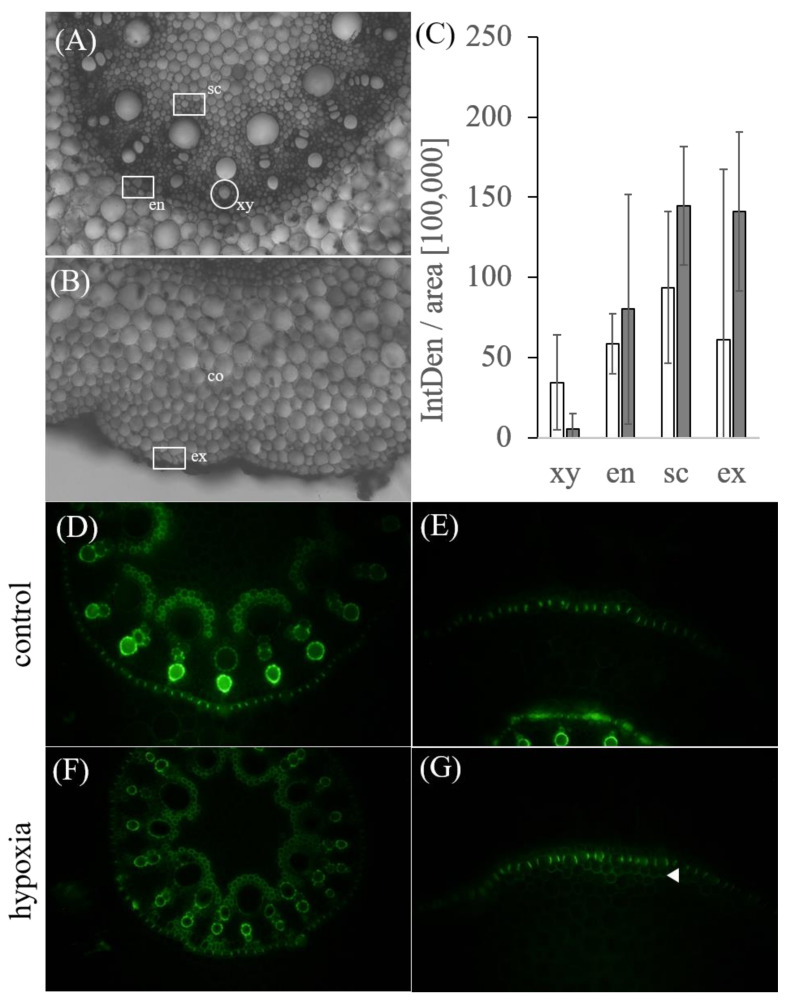
Visualization of suberin in the mature zone of primary root cross-sections from control and hypoxia-stressed maize plants. Control and 24 h hypoxia-stressed maize roots were cross-sectioned by hand and stained with the fluorescent berberine–aniline blue to detect suberin. Images were taken with 10x magnification using an Olympus BHS fluorescent microscope. (**A**,**D**,**F**) Shown is an overview of the central cylinder with vascular bundles and (**B**,**E**,**F**) the cortex and exodermis of maize root transversal section in brightfield image (**A**,**B**) and fluorescent image (**D**–**G**). (G) White arrow indicates an additional suberin layer beneath the exodermis in stressed samples. (**C**) Intensity measurements of endodermis (en), exodermis (ex), vascular sclerenchyma cells (sc) and xylem vessels (xy) were calculated as intensity per area (IntDen/area) for controls (white columns) and stressed samples (grey columns). Error bars indicate standard deviation for three to five biological replicates.

Berberin–aniline staining, quantified as intensity per area (IntDen/area), revealed a decreased intensity of suberin in xylem vessels (controls with 34.52 ± 29.71 and stressed with 5.60 ± 9.29 IntDen/area in 100,000). Contrary to this, there is a slightly, but not significant, increased intensity in vascular sclerenchyma cells (controls with 93.57 ± 47.36 and stressed with 144.87 ± 37.06 IntDen/area in 100,000), endodermis (controls with 58.60 ± 18.89 and stressed with 80.10 ± 71.88 IntDen/area in 100,000), and exodermis cells (controls with 61.33 ± 105.91 and stressed with 141.03 ± 49.52 IntDen/area in 100,000) (Figure 8A). A closer look at the exodermis showed an additional suberin layer beneath the exodermis in stressed samples (Figure 8B).

## 3. Discussion

For the first time, this study shows the induction of PM-bound class III peroxidases in maize roots by hypoxia. Furthermore, we show that the differential regulation of specific class III peroxidases caused cell wall modifications in response to hypoxia and present evidence for the interaction of PM bound class III peroxidases with *Zm*RbohB and *Zm*Rboh4.

### 3.1. Hypoxia-Responsive Class III Peroxidases 

The transcriptome analyses of maize roots under hypoxia revealed that most of the differentially expressed peroxidase genes were soluble proteins (70%), localized either in the apoplast or bound to cell walls (Figure 1). Only two of these soluble peroxidases (*zmprx06* and *zmprx117*) were upregulated. Although gene regulation may be different in root and leaf, *Zm*Prx06 was found in a leaf soluble fraction together with other peroxidases [20]. For this fraction, guaiacol peroxidase activity increased by hypoxia. In roots, the majority of soluble peroxidases (*n* = 10) was downregulated. For membrane-bound peroxidases, the transcript of a putative peroxidase (*zmprx135*) of the endoplasmic reticulum was upregulated, whereas putative PM peroxidase transcripts were either not differentially regulated or downregulated (*zmprx109* and *zmprx140*) (Figure 1C). These peroxidases have not been investigated on the protein level. In silico analyses of *zmprx135* predicted a function related to abscisic acid and heat stress [36].

Fractionation of the root samples showed an increase in membrane-bound peroxidase activities and a decrease in soluble peroxidase activity by hypoxia (Figure 2A). This result fits well with the downregulation of several soluble peroxidase genes (Figure 1A). The increase of peroxidase activity in microsomal fractions (Figure 2A) could be partially explained by the upregulation of *zmprx135*. This assumption will need further proof for the gene product.

However, peroxidase abundance appeared to be higher for the soluble fraction compared to membrane fractions (Figure 2B). This observation may partially depend on the detergent used for solubilization of membrane proteins. Solubilization of PM by CHAPS revealed the maximal peroxidase activity of non-stressed samples for maize seedlings [35]. CHAPS-insoluble membranes still showed guaiacol peroxidase bands with *Zm*Prx81 and *Zm*Prx85 that were more intense in stressed samples. Additionally, *Zm*Prx01 and *Zm*Prx70 were found only in stressed samples (Figure 4C,D). It has been shown that CHAPS and Triton X-100 selectively extract glycerophospholipids and some proteins, whereas the resulting insoluble membranes are strongly enriched in sphingolipids and cholesterol [37]. Identification of these peroxidases in CHAPS-insoluble membranes supports not only a strong interaction of the proteins with the PM [34] but also a localization of the enzymes in microdomains [23].

Both, gel-free and gel-based peroxidase analyses of PM revealed significant increases in the abundance of *Zm*Prx03, *Zm*Prx24, *Zm*Prx81, and *Zm*Prx85 (Figure 3 and Figure 4). None of these peroxidases appeared differentially regulated on the transcriptional level, using RNA Seq analyses (Supplementary Materials Table S1A). The observed downregulation of *zmprx109* and *zmprx140* (Figure 1C) was in agreement with the fact that these putative PM peroxidases could not be detected on the protein level.

To verify the gene regulation of the identified peroxidases as well as PM peroxidases that were already identified in primary maize roots [34], RT-qPCR was performed. The results revealed a significant upregulation of *zmprx01, zmprx24,* and *zmprx85* and a tendency of higher expression levels for *zmprx03, zmprx70,* and *zmprx81* in stressed samples whereas *zmprx66* was not differentially regulated by hypoxia (Figure 5). A difference between RNA Seq and RT-qPCR was observed for smaller genes with fewer exons and lower expression [38]. Hypoxia-induced upregulation of *zmprx01* confirmed results of chip-based analyses [21]. The higher expression of *zmprx01* did not correlate with changes in abundance of *Zm*Prx01 in gel-free or gel-based proteome analyses (Figure 3 and Figure 4). The protein was not found in LC–MS/MS in contrast to *Zm*Prx03, *Zm*Prx24, *Zm*Prx81, and *Zm*Prx85. A possible reason might be the higher amount of protein (250 µg) used for 2D-PAGE compared to LC–MS/MS (100 µg). In gel-based analyses, *Zm*Prx01 was not only detected in spots 4 and 5 but also in CHAPS-insoluble membranes (Figure 4). Additionally, *Zm*Prx01 revealed a stronger interaction with the PM in the hypoxia-stressed samples compared to the control. These results complicate a quantitative analysis of *Zm*Prx01 compared to the other peroxidases.

### 3.2. Membrane Protection and Aerenchyma Formation

The observed higher level of hydrogen peroxide in hypoxia-stressed root samples confirmed a production of ROS that lead to oxidative stress and lipid peroxidation [6]. A 5.9-fold upregulation of *zmrboh10* was found (Supplementary Materials Table S1B). This isoform has a function in ROS production and aerenchyma formation in maize roots [13] which fits nicely with the observed aerenchyma in hypoxia-stressed root cross-sections (Figure 6). The higher expression levels of expansins and the downregulation of *zmccoaomt* correlate with the cell wall loosening and degradation during aerenchyma formation (Supplementary Materials Table S1C). The consumption of hydrogen peroxide by hypoxia-induced PM peroxidases (*Zm*Prx01, *Zm*Prx03, *Zm*Prx24, *Zm*Prx70, *Zm*Prx81, and *Zm*Prx85) regulates not only the level of ROS but also protects the membranes of neighbor cells against oxidative stress and lipid peroxidation. Additionally, cell lysis will release NADH that can react with apoplastic and cell wall class III peroxidases and thereby increase ROS production and cell wall loosening [6,39].

### 3.3. Peroxidase–Rboh Interaction and Cell Wall-Remodeling

Protein assemblies may not be destructed by native IEF or non-denaturing SDS-PAGE as used in the present study and could explain the high molecular masses (100–290 kDa) of the peroxidase spots in 2D-PAGE (Figure 4). The observed co-separation of *Zm*RbohB and *Zm*Rboh04 isoforms with at least *Zm*Prx01, *Zm*Prx03, and *Zm*Prx24 in 2D-PAGE (Supplementary Materials Table S3) suggests an interaction between these proteins.

Transmembrane helices were predicted for *Zm*Prx01, *Zm*Prx70, *Zm*Prx81, and *Zm*Prx85 (Supplementary Materials Table S4). The co-localization of peroxidases with Rboh in functional microdomains could present a mechanism for the fine-tuning of ROS levels and may prevent lipid peroxidation not only under stress conditions [34]. In contrast to the peroxidases, none of the putative interaction partners (*zmrbohB* isoforms, *zmrboh04*) were differentially regulated by hypoxia (Supplementary Materials Table S1B). Opposite results have been found for tomato (*Solanum lycopersicum* L.) roots that showed a strong upregulation of *slrbohB* [14]. In contrast to maize, tomato is a flooding-sensitive species, which may explain this observation.

Protein assemblies may also explain the occurrence of *Zm*Prx03 and *Zm*Prx24 at the PM. These peroxidases appeared to be soluble because transmembrane helices were not predicted for these proteins (Supplementary Materials Table S4). A soluble class III peroxidase in *Arabidopsis thaliana* (L.) Heynh. (*At*Prx64) was shown to interact with *At*RbohF via a Casparian strip protein and a dirigent-like protein [40]. This protein assembly has a function in lignification during Casparian strip formation in the endodermis. Although homologs of *At*Prx64 were not found in maize, the soluble peroxidases (*Zm*Prx03, *Zm*Prx24) identified in PM may have comparable functions in cell wall processes during hypoxia. Further, higher abundances of dirigent proteins in hypoxia-stressed samples support an interaction between the upregulated peroxidases and *Zm*Rboh (Supplementary Materials Table S2B). To confirm this assumption, a detailed biochemical characterization will be necessary for the future.

Although the data at hand did not allow a detailed discussion of the specific functions of hypoxia-induced peroxidases yet, the higher abundances of *Zm*Prx01, *Zm*Prx03, *Zm*Prx24, *Zm*Prx70, *Zm*Prx81, and *Zm*Prx85 in PM of stressed samples (Figure 3 and Figure 4) point to a function of these peroxidases in cell wall remodeling. Other class III peroxidases *(zmprx06*, *zmprx117*, and *zmprx135),* that were found to be differentially regulated by hypoxia, indicate participation in these processes as well.

Deposition of lignin was observed in hypoxia-stressed maize roots by specific stains (Figure 7A–C). Due to a lack of biochemical characterization of hypoxia-responsive peroxidases, a statement on specific peroxidases involved in these processes may be incomplete. Although the higher expression level of *zmprx70* was not significant (Figure 5), its gene product was detected with higher abundance in microdomains of stressed samples (Figure 4). Biochemical characterization of the partially purified *Zm*Prx01 and *Zm*Prx70 showed a significant increase of guaiacol peroxidase activity in the presence of ferulic acid [35]. For both peroxidases, the preferred substrate—in the absence of guaiacol—was the lignin precursor coniferyl alcohol. Thus, at least *Zm*Prx01 and *Zm*Prx70 should be involved in the observed lignification of hypoxia-stressed roots. This hypothesis was further supported by the upregulation of key enzymes (*zmces* and *zmcad*) of the phenylpropanoid pathway (Supplementary Materials Table S1C) and the higher abundances of dirigent proteins and *Zm*FLA10 (Supplementary Materials Table S2B). Fasciclin-like arabinogalactan proteins precede lignification [41]. Association of *Zm*Prx01 and *Zm*Prx70 with *Zm*CAD1 was suggested by the Search Tool for the Retrieval of Interacting Genes/Proteins (STRING) database [42]. Cinnamyl alcohol dehydrogenase facilitates the substrates for the final peroxidase dependent step of lignin-monomer formation [26]. A function of *Zm*Prx01 and *Zm*Prx70 in monolignol biosynthesis fits nicely with the increase of their abundances observed by fungal elicitors [19]. The function of monolignol biosynthesis plays a crucial role in cell wall apposition-mediated defense against pathogens [43].

Chlorine–zinc–iodine staining revealed lower amounts of cellulose in cross-sections of stressed samples (Figure 7D). This observation matches the downregulation of several cellulose synthases (Supplementary Materials Table S1C), but appear to disagree with the upregulation of *zmcesa*5. Cellulases were also downregulated after 24 h. Although *zmgdpdl3* (A0A1D6KIK2, C0PL13) was significantly upregulated in the RNA Seq experiment, its gene product was not detected in the PM. Abundances of the two *Zm*GDPDL (A0A1D6HBU2, C0PGU8), identified in PM, showed a weak increase compared to controls. Transcripts of these *Zm*GDPDL (C0PGU8, A0A1D6HBU2) were either not differentially regulated or downregulated. Thus, the lower expression of several *zmgdpdl* further supports the decrease in cellulose. Knockouts of this enzyme revealed a lower content of crystalline cellulose [44].

Besides the cellulose, cell walls of grasses contain high amounts of hemicellulose (55%) consisting of arabinoxylans, xyloglucan, and mixed-linked glucans [45]. The higher levels of hydrogen peroxide in hypoxia-stressed samples support a generation of diferulates by *Zm*Prx03, *Zm*Prx24, *Zm*Prx81, or *Zm*Prx85 and thereby a crosslinking of arabinoxylans and lignin by phenolics [31,46]. In maize suspension-cultured cells, it has been demonstrated that a decrease in cellulose content can be compensated by the deposition of lignin-like polymers and a network of highly crosslinked feruloylated arabinoxylans [31].

As shown in Figure 8, quantitative analyses of the berberin–aniline staining revealed a weak increase of suberin in vascular sclerenchyma cells, endodermis and exodermis cells. Peroxidases catalyze the oxidation of cinnamyl alcohols before their polymerization by a peroxidase/hydrogen peroxide-mediated process during suberin formation [27,47,48]. However, *zmhht* that is related to suberin synthesis was downregulated by hypoxia. Enstone and Peterson [49] showed that maize roots grown in hydroponics had significantly fewer suberin lamellae in endodermis and exodermis compared to plants grown in other substrates (e.g., vermiculite). Additionally, induction of exodermal Casparian bands or suberin lamellae failed in the lateral roots of maize grown in hydroponics [50]. Although the formation of a ROL barrier by peroxidases identified was supported by the deposition of lignin and suberin in hypodermal/exodermal cell layers in hypoxia-stressed samples (Figure 7 and Figure 8) and a possible crosslinking of arabinoxylans and lignin, the function of the peroxidases in these processes will need further investigations.

## 4. Materials and Methods

### 4.1. Plant Material and Growth Conditions

Maize caryopses (*Zea mays* L. cv. Gelber Badischer Landmais, Saatenunion, Hannover, Germany) were soaked in fully desalted water for 4–6 h and sterilized with 3% H_2_O_2_ for 10 min. In trays, sterilized with 70% ethanol, the kernels were placed onto and covered with wetted germination tissue. The trays were covered with aluminum foil and stored in dark for four days at 26 °C. The seedlings were transferred into 9 L boxes filled with hydroponic culture medium (5.25 mM KNO_3_, 7.75 mM Ca(NO_3_)_2_ 4H_2_O, 4.06 mM MgSO_4_ 7H_2_O, 1.0 mM KH_2_PO_4_, 100 µM Fe(III)-EDTA, 46 µM H_3_BO_4_, 9.18 µM MnSO_4_ H_2_O, 5.4 µM ZnSO_4_ 7H_2_O, 9.0 µM CuSO_4_ 7H_2_O, 2.0 µM Na_2_MoO_4_ 2H_2_O, pH 5.5) which was changed once after ten days. Culturing was performed in a climate chamber (light source: Philips SGR 140 with Philips SON-T Agro 400 W sodium vapor lamp, about 400–500 µmol m^−2^ s^−1^, 12 h day/night, temperature: 22 °C day/18 °C night), the medium was oxygenated by KOH washed air (compressor type LK60, OSAGA, Glandorf, Germany). After 14 days of culturing, oxygenation was stopped and hypoxia stress was induced by preventing the oxygen supply with 500 mL commercially available rape oil that led to a reduction of oxygen from 21% to 3.5 ± 0.5% after 24 h of stress. The pH thereby stayed stable at 6.8 ± 0.5 to 6.3 ± 0.7 within 24 h of stress induction. Contrary to these stressed plants, control plants were continuously supplied with air (21% oxygen). After 24 h, the roots were harvested between 9 and 10 a.m. (CET). Adhered oil was removed from the roots by washing with 0.1% Triton X-100 for 15–30 s.

### 4.2. Preparation of Subcellular Fractions

Maize roots were washed (3 mM KCl, 0.5 mM CaCl_2_, 0.125 mM MgSO_4_) and homogenized (0.25 M sucrose, 50 mM HEPES, 5 mM Na_2_-EDTA, pH 7.5, supplied with 1 mM dithiothreitol and 1% polyvinylpolypyrrolidone), using a Waring blender 7011HS (Co. Waring, Stamford, CT, USA). The homogenate was filtered through a nylon net (125 µm mesh, Co. Hydro-Bios, Kiel, Germany) and 1 mM phenylmethylsulfonyl fluoride was added (=total fraction). After the first centrifugation at 10,000× *g* for 10 min at 4 °C (Avanti J-E centrifuge, rotor type JA-14, Beckman Coulter, Krefeld, Germany), the supernatant was centrifuged at 48,000× *g* (Avanti J-E centrifuge, rotor type JA-25.50, Beckman Coulter, Krefeld, Germany) for 30 min at 4 °C, which resulted in a supernatant with mainly cytosolic, soluble components, and a microsomal pellet. The proteins of the soluble fraction were precipitated with 90% saturated (662 g/L) ammonium sulfate overnight at 4 °C, pelleted at 15,000× *g* (Avanti J-E centrifuge, rotor type JA-25.50, Beckman Coulter, Krefeld, Germany) for 20 min at 4 °C and resolved (0.25 M sucrose, 50 mM HEPES, pH 7.0). The microsomal pellet was resolved in phase buffer (0.25 M sucrose, 5 mM KCl, 5 mM phosphate buffer, pH 7.8) and were used either directly for the following aqueous polymer two-phase partitioning or stored at −76 °C until further use. Plasma membranes were isolated from the microsomal fractions by 36 g phase systems (0.25 M sucrose, 5 mM phosphate buffer, pH 7.8, 5 mM KCl, and 6.5% Dextran T500, 6.5% polyethylene glycol 3350) [51]. Proteins were quantified by using Pierce^TM^ Bovine Serum Albumin Standard (BSA, Co. ThermoFisher Scientific, Waltham, MA, USA, from 2 to 20 μg) for calibration [52].

### 4.3. Hydrogen Peroxide Assay

For H_2_O_2_ determination, root tissue of three controls and three 24 h hypoxia-stressed maize plants was ground with liquid nitrogen (fresh weight about 1.3 g) and homogenized in 4 mL buffer (250 mM sucrose, 50 mM HEPES pH 6.8, 1 mM dithiothreitol, and 1% polyvinylpolypyrrolidone) per g fresh weight and centrifuged at 16.000× *g* for 10 min at 4 °C (rotor Sorvall #3325B, Heraeus Biofuge fresco, ThermoScientific). Hydrogen peroxide was estimated by the ferric-xylenol orange assay [53,54]. For short, two reagents (reagent A with 25 mM (NH_4_)_2_Fe(SO_4_)_2_, 110 mM HClO_4_, and reagent B with 125 mM xylenol orange, 100 mM sorbitol) were mixed at a ratio of 1:100. The supernatant of the samples (200 µL) was mixed with xylenol orange solution (1 mL) and incubated in the dark for 30 min. The absorbance was measured with a dual-beam UV/Vis-Spectrophotometer (Type UV-1800, Co. Shimadzu, Hamburg, Germany) at 560 nm. For quantification of H_2_O_2_, a dilution series of H_2_O_2_ was produced in oxygen-depleted water which resulted in a calibration curve with a linear range up to 625 µmol H_2_O_2_.

### 4.4. Peroxidase Activity

The activity of class III peroxidases was determined in different fractions (total, microsomes, soluble, and PM). The assay contained 775 µL 25 mM sodium acetate buffer pH 5.0, 100 µL 0.3% H_2_O_2_ (Co. AppliChem, Darmstadt, Germany), 100 µL 89 mM guaiacol (Co. Merck KGaA, Darmstadt, Germany), and 25 µL protein sample with different protein amount (0.02–50 µg). The turnover of guaiacol to tetraguaiacol (ε_470 nm_ = 26.6 mM^−1^·cm^−1^) was measured for 2 min at 470 nm with the UV-1800 spectrophotometer. Values given were from six biological and three technical replicates per sample. The buffer and the two substrates served as a reference.

### 4.5. Gel-Based Analyses and Mass Spectrometry

Modified SDS-PAGE with 11% polyacrylamid gels was used for the separation of subcellular fractions. Therefore, samples were mixed with 4 x non-reducing loading buffer (500 mM Tris-HCl pH 6.8, 80% (*w*/*v*) glycerol, 0.08% (*w*/*v*) SDS, bromophenol blue; [51]) and separated 10 min at 80 V and about 120 min at 120 V.

For two-dimensional polyacrylamide gel electrophoresis (2D-PAGE), washed PM (250 µg total protein content) were pelleted at 105,000 *g* for 30 min at 4 °C, solubilized in 3x IEF loading buffer (8% ampholytes, 8% CHAPS, 40% glycerol, 3 M urea) on ice for 1 h and centrifuged again. Afterwards, the remaining pellet was solubilized with 7:1 SB12 on ice for 2 h and centrifuged at 105,000× *g* for 45 min or 13,000× *g* for 60 min at 4 °C [23]. The first dimension was performed with native IEF gels (2% CHAPS, 3 M urea, 7.5% acrylamide, 2% ampholytes pH 5–8 and 8–11) with 20 mM NaOH (cathode buffer) and 10 mM phosphoric acid (anode buffer) in an electric gradient (12 h 30 V, 2 h 100 V, 1.5 h 250 V, 1 h 300 V) at 4 °C. The pH of the 0.5 cm thick gel pieces was measured after the run. The sample-loaded gel lanes were equilibrated (125 mM Tris, 1% SDS, 10% glycerol, pH 8.8) at 4 °C for 1 h and transferred to a 4–18% polyacrylamide gradient gel for the second dimension under non-reducing conditions. This gel electrophoresis was performed at 4 °C with 30 mA per gel for 10 min at 80 V and about 120 min at 150–200 V. After the run, gels were stained with guaiacol (0.5% (*v*/*v*) in 50 mM sodium acetate buffer pH 5.0 and 0.5% H_2_O_2_) or 3,3′,5,5′-tetramethylbenzidine (4.7 mM TMB, 30% methanol in 50 mM sodium acetate buffer pH 5.0 and 0.1% H_2_O_2_). The intensity of single spots was quantified by using Image J (Image J software version 1.53a, Bethesda, MD, USA). The determination of pI and MW was performed by using pH and protein standard [51]. TMB stained protein spots were cut out and in-gel digestion of proteins and liquid chromatography MS (LC–MS/MS) was done [55]. LC–MS/MS data were processed with Proteome Discoverer 2.0 (Thermo Scientific, Bremen, Germany). Identification of the proteins from the MS/MS spectra was performed with the search engine Sequest HT, using the MaizeGeneDatabase (https://www.maizegdb.org/) and the Peroxibase (http://peroxibase.toulouse.inra.fr/). For the searches, the following parameters were applied: precursor mass tolerance: 10 ppm and fragment mass tolerance: 0.2 Da. Two missed cleavages were allowed. Carbamidomethylation on cysteine residues as a fixed modification and oxidation of methionine residues as a variable modification was used for the search. Peptides with a false discovery rate of 1%, using Percolator, were identified. At least two unique peptides per protein were used as a condition for reliable identification. Peroxidase nomenclature was used in accordance with Peroxibase.

### 4.6. Gel-Free Peroxidase Analyses

Plasma membrane preparation and further MS analyses were done according to previous studies [56]. PM of three stressed and three control plants were enriched and prepared before MS analyses as described as followed: 100 µg total PM protein were washed (250 mM sucrose, 50 mM HEPES, 150 mM KCl, 0.01% Triton X-100) for 30 min, then pelleted for 1 h at 13,000× *g* at 4 °C. The pellet was incubated in 200 µL of solubilization buffer (125 mM Tris-HCl pH 6.5, 2% SDS, 5% mercaptoethanol, 6 M urea) for 1 h at room temperature and centrifuged again at 13,000× *g* for 60 min. Proteins in the resulting supernatant were precipitated with 1.8 mL methanol:chloroform (4:1) at −20 °C overnight, centrifuged, and washed three times in 0.5 mL pure methanol by centrifugation at 13,000× *g* for 20 min. The washed pellet was dried for 30 min, resuspended in 50 µL digestion buffer (200 mM NH_4_CO_3_ pH 8.5, 8 M urea, 10% acetonitrile (ACN)) and incubated in addition of 0.1–0.5 µg lysin C at 37 °C for 16–18 h. Then, the sample was diluted 1:3 in 10% ACN, 10 µL of trypsin beads were added and incubated at 37 °C for 16–24 h for hybridization. This digestion was stopped by adding three times volume of 0.3% heptafluorobutyric acid and trypsin beads were removed by centrifugation. Peptides were washed and dried, using ZipTips (Co. Agilent Technologies, Santa Clara, CA, USA) according to the manufacturer´s protocol. Peptides were dissolved in 2% ACN, 0.1% formic acid. In random order 1 µg was applied on a C18 column (15 cm, 50 mm column, PepMapR RSLC, Thermo Scientific, 2 mm particle size) for separation during a 90 min gradient at a flow rate of 300 nL min^−1^. Measurement was done on an LTQ-Orbitrap Elite (Thermo Fisher Scientific, Bremen, Germany) with the following settings: full scan range 350–1800 m/z, max 20 MS2 scans (activation type CID), repeat count 1, repeat duration 30 s, exclusion list size 500, exclusion duration 60 s, charge state screening enabled with a rejection of unassigned and +1 charge states, minimum signal threshold 500. Proteins were identified and quantified as described earlier [57], using a UniprotKB FASTA download for *Zea mays* (UP000007305) and the software MaxQuant v1.6.5.0 with the following parameters: first search peptide tolerance 20 ppm, main search tolerance 4.5 ppm, ITMS MS/MS match tolerance 0.6 Da. A maximum of 3 of the following variable modifications were allowed per peptide: oxidation of methionine and acetylation of the N-term. A maximum of two missed cleavages were tolerated. The best retention time alignment function was determined in a 20 min window. Identifications were matched between runs in a 0.7 min window. An FDR cutoff at 0.01 (at Peptide Spectrum Match and protein level) was set with a reversed decoy database. A minimum of seven amino acids was required for the identification of peptides and at least two peptides were required for protein identification. The resulting data matrix was filtered so that there are label-free quantifications (LFQ) in at least one of the treatments (control and stressed) and more than four replicates (biological and/or technical replicates). Missing values that appear due to low abundant proteins or an oversupply of peptides during MS run were corrected with COVAIN [58]. The LFQ intensities (the normalized intensities) of the control and stressed samples were averaged of three biological and two technical replicates. Standard deviation and Student´s *t*-test were used to determine significant changes. The stressed samples were normalized to the controls. The obtained ratios show either increase (ratio > 1.05) or decrease (ratio < 0.95) of the proteins on a comparison pair (stressed versus control).

### 4.7. Isolation of Total RNA

Stressed and control maize roots of three biological replicates were harvested after 24 h. For each biological replicate, at least five plants were pooled. The roots were ground with a mortar and pestle, using liquid nitrogen to get a very fine powder (about 0.3 g fresh weight). Total RNA isolation from this powder was done with the NucleoSpin^®^ RNA Plant and Fungi Kit (Co. Macherey-Nagel, Düren, Germany). For RNA Seq analyses, a final step of ethanol precipitation with 1/10th volume of 3 M sodium acetate pH 5.2 and three volumes of 100% ethanol absolute was added before delivering the samples to Macrogen Inc. (Seoul, South Korea) for further analyses.

### 4.8. Quality Control and RNA Sequencing (RNA Seq)

Quality control (QC) and analyses of the total RNA samples were done by Macrogen Inc. (Seoul, South Korea). QC analyses for verifying the quantity and quality of the RNA samples were performed by using agarose gel electrophoresis and an Agilent Technologies 2100 Bioanalyzer (Agilent Technologies, Santa Clara, CA, USA). Six high-quality RNA samples with an RNA Integrity Number (RIN) value greater than or equal to seven were used for cDNA library construction. Sequencing was done, using Illumina Sequencing. RefGen_v4 of maize was used as a reference gene (ftp://ftp.ncbi.nlm.nih.gov/genomes/all/GCF/000/005/005/GCF_000005005.2_B73_RefGen_v4/)

### 4.9. Quantitative Reverse-Transcription Polymerase Chain Reaction (RT-qPCR)

Expression levels of proteins, identified by MS, were verified by RT-qPCR. The concentration and purity of the isolated total RNA were determined with a Nanodrop spectrophotometer (Fisher Scientific GmbH, Schwerte, Germany) and agarose gel electrophoresis (1.5% agarose in 1x TAE (40 mM Tris, 20 mM acetic acid, 1 mM EDTA), run 60 V 3 h). The cDNA was prepared from 100 ng of total RNA with the First Strand cDNA Synthesis Kit (Co. Fisher Scientific GmbH, Schwerte, Germany) according to the manufacturer´s protocol. Efficiencies of the primers were checked first with PCR (7 min 95 °C, 30–35 cycles of 20 s 95 °C, 30 s 60 °C, 30 s 72 °C, and finally 7 min 72 °C), using the Maxima Hot Start Kit (Co. Thermo Scientific, Massachusetts, USA) followed by agarose gel electrophoresis (1% agarose in 1x TAE, run 60 V) and second with RT-qPCR (5 min 95 °C, 40 cycles of 10 s 95 °C and 30 s 60 °C terminating in a melting curve from 65 to 95 °C with 0.5 °C s^−1^ steps), using Quantifast SYBR green PCR kit (Qiagen GmbH, Hilden, Germany) and the CFX 96 Cycler (CFX96 Touch system, Bio-Rad, Munich, Germany). To analyse specific maize peroxidases (ZmPrx), a set of primers were designed (Eurofins Genomics Germany GmbH, Ebersberg, Germany, Table 1). As a housekeeping gene, *Zea mays* translational elongation factor EF-Tu (*zmtufM*, AF264877.1, Q9FUZ6) was used. For statistical analysis, RT-qPCR was performed twice for three biological replicates of each treatment and compared to the housekeeping gene, using the CFX manager software version 3.1 (Co. Bio-Rad, Hercules, USA).

### 4.10. In Vivo Cell Wall Staining

Handmade cross-sections of maize primary roots (24 h hypoxia-stressed and controls; mature differentiation zone) were prepared with a razor blade, stained with different methods, and imaged. A Leica DM500 binocular microscope (10x objective #13613241 and 40x objective #13613242, Leica, Wetzlar, Germany) and an Olympus BHS fluorescent microscope (10x SPLAN Apo objective, Olympus “B” dichroic mirror (DM500) and EY455 excitation filter) with excitation from 455 to 490 nm and emission LP at about 515 nm) were used. For Mäule staining, sections were placed in 1% KMnO_4_ solution for 5 min, and then washed 3x with water. After 30 min incubation in fresh prepared 1 N HCl solution, sections were washed again, and 1 M Tris-HCl pH 8.0 was added [59]. Mäule staining results in a positive deep-red colored reaction produced by 3-methoxy-o-quinone structures generated from syringyl lignin monomers (derived from sinapic acid) or a negative yellow reaction. The latter indicates the localization of guaicyl lignin monomers (derived from ferulic acid) [59,60,61]. For Wiesner stain, sections were incubated in 3% phloroglucinol-in-ethanol solution for 20 min. After adding 37% HCl the sections were directly imaged. “Using the phloroglucinol reagent, a distinction can be made between (I) aldehydes (intense orange-red colour), (II) anethole, asarones, isosafrole (no colour) and (III) the group of eugenol, methyleugenol, myristicin and safrole (pink)” [62]. Yellow-to-red colors develop with certain compounds containing aldehydes. A strong orange staining is determined when a reaction with cinnamic aldehydes or cinnamic alcohols occurs [63,64]. Phloroglucinol-HCl (Wiesner reagent) reacts with the cinnamaldehyde groups in lignin, resulting in a pink color of lignified cell walls [61]. Etzold staining is a simultaneous staining with fuchsin, chrysoidin, and Astra blue (FCA). A ready-to-use staining solution is commercially available (Co. Morphisto GmbH, Frankfurt am Main, Germany) and added directly onto the sections. Non-ligneous cells, cell walls, and phloem show blue, ligneous cell walls, and xylem show red color. A ready-to-use solution of chlorine–zinc–iodine according to Behrens (Co. Morphisto GmbH, Frankfurt am Main, Germany) was used for the detection of cellulose. A positive reaction results in blue to violet color. To detect suberines [65,66], sections were incubated in dark for 1 h in 0.1% berberine hemisulphate, then washed and incubated 30 min in 0.5% anilin blue, then washed again and imaged. Shading correction of the fluorescent images was done with Image J plugin BaSiC [67], using 2.5% Lucifer Yellow as a flat field image.

## 5. Conclusions

For the first time, regulation, abundance, and activity of hypoxia-responsive class III peroxidases of the PM were studied. The data at hand revealed functions in (i) cell-wall loosening and membrane protection during aerenchyma formation; and (ii) lignification (*Zm*Prx01, *Zm*Prx70), suberization, and cell wall crosslinking during hypoxia-induced cell wall remodeling. To clarify specific functions of hypoxia-responsive peroxidases (*Zm*Prx01, *Zm*Prx03, *Zm*Prx24, *Zm*Prx70, *Zm*Prx81, and *Zm*Prx85), future research needs to be focused on peroxidase–Rboh interaction and biochemical characterization of these peroxidases. Due to the significant upregulation of *zmpr01*, *zmprx24,* and *zmprx85* by hypoxia, these peroxidases are suitable hypoxia-specific stress marker candidates.

## Figures and Tables

**Table 1:** Primer sequences in 5′-3′-orientation for RT-qPCR.

Name of Peroxidase	Forward Primer	Reverse Primer
*zmprx01*	ACTTGTTCAAGGCCAAGGAG	TTCGTGCTTGTGTTCCAGAC
*zmprx03*	TCAAGATGGGGCAGATCGAG	ACTCCAGTGAATCCTGATGGG
*zmprx24*	GGCTCATCCGCATCTTCTT	TGGTTGGGTACCTCGATCT
*zmprx66*	CGACATGGTTGCACTCTCAG	CGAAGGCGGAGTTGATGTTG
*zmprx70*	CCACCTCCATGACTGCTTTG	TTCGGATTAGCGGTCTGCTC
*zmprx81*	CAGGAGGATGACTTCGCCAG	CCGTTGTAGGGTCCCTGATG
*zmprx85*	GACGCTGAGGAAGAACAAGG	CTGGTCGAAGAACCACCAG
*zmtufM*	CGCAGTTGATGAGTACATCC	AACACGCCCAGTAACAACAG

## Supplementary Materials

Supplementary Materials can be found at https://www.mdpi.com/1422-0067/21/22/8872/s1. Figure S1: Western blot. Figure S2: Abundance of guaiacol peroxidases under hypoxia stress. Figure S3: Biological and technical replicates of 2D-PAGE pH 9–11. Figure S4: Biological and technical replicates of 2D-PAGE pH 5–9. Table S1A: RNA Seq analyses of class III peroxidases. Table S1B: RNA Seq analyses of RBOH. Table S1C: RNA Seq analyses of cell wall-related genes. Table S2A: Mass spectrometry analyses of class III peroxidases. Table S2B: Mass spectrometry analyses of cell wall-related proteins. Table S3: Mass spectrometry analyses of the peroxidases. Table S4: In silico prediction of the identified class III peroxidases.

## Abbreviations

2D	two-dimensional
ACN	acetonitrile
*At*	*Arabidopsis thaliana*
BSA	bovine serum albumin
C	control sample
CAD	cinnamyl alcohol dehydrogenase
CCoAOMT	caffeoyl-CoA *O*-methyltransferase 1
cDNA	copy desoxyribonucleic acid
CESA	cellulose synthase
CET	Central European Time
CHAPS	3-[(3-cholamidopropyl)dimethylammonio]-1-propanesulfonate
co	Cortex
Cox2	cytochrome c oxidase
CSE	caffeoylshikimate esterase
*Cv*	Cultivar
DEGs	differentially expressed genes
DIR	Dirigent
ε_470 nm_	extinction coefficient at 470 nm
ECL	enhanced chemiluminescence
EDTA	ethylenediaminetetraacetic acid
EF	elongation factor
en	Endodermis
ER	endoplasmic reticulum
ex	Exodermis
EXP	Expansin
EXPL	expansin-like
FCA	fuchsin, chrysoidin, and Astra blue
FDR	false discovery rate
FLA	fasciclin-like arabinogalactan
FPKM	fragments per kilobase million
GDPD	glycerophosphodiesterase
GDPDL	glycerophosphodiester phosphodiesterase-like
H	hypoxia-stressed sample
H^+^-ATPase	PM specific H^+^ATPase
HEPES	4-(2-hydroxyethyl)-1-piperazineethanesulfonic acid
HHT	hydroxycinnamoyl-CoA:ω-hydroxyacid *O*-hydroxycinnamoyltransferase
hy	hypodermis
IEF	isoelectric focusing
IntDen/area	Integrated Density per area
kDa	kilodalton
LC–MS/MS	liquid chromatography mass spectrometry
LFQ	label-free quantifications
micro	microsomes
MS	mass spectrometry
MW	molecular weight
NADH	nicotinamide adenine dinucleotide
NCBI	National Center for Biotechnology Information
PAGE	polyacrylamid gel electrophoresis
ph	phloem
pI	point isoelectric
PM	plasma membrane
ppm	parts per million
Prx	class III peroxidases
QC	quality control
Rboh	respiratory burst oxidase homologs
RIN	RNA integrity number
RNA	ribonucleic acid
RNA Seq	RNA sequence analyses
ROL	radial oxygen loss
ROS	reactive oxygen species
RT	room temperature
RT-qPCR	real-time quantitative polymerase chain reaction; quantitative reverse-transcription polymerase chain reaction
SB12	n-dodecyl-*N*, *N*-dimethyl-3-ammonio-1-propanesulfonate
sc	vascular sclerenchyma cells
SDS	sodiumdodecylsulfate
STRING	Search Tool for the Retrieval of Interacting Genes/Proteins
TAE	tris-acetate-EDTA
TMB	3,3′,5,5′-Tetramethylbenzidine
Tris	tris(hydroxymethyl)aminomethane
Triton X-100	2-[4-(2,4,4-trimethylpentan-2-yl)phenoxyl] ethanol
tufM	thermo unstable translation elongation factor, mitochondrial
V-PPase	pyrophosphate-energized vacuolar membrane proton pump 1
xy	xylem
*Zm*	*Zea mays*
