# Supplementary material for: Hypoxia-Responsive Class III Peroxidases in Maize Roots: Soluble and Membrane-Bound Isoenzymes
Source: Int J Mol Sci. 2020 Nov 23;21(22):8872. doi: 10.3390/ijms21228872 (PMC7700428; doi:10.3390/ijms21228872)
Supplement: Supplementary file 1 [file ijms-21-08872-s001.zip › Supplementary data.pdf]

International Journal of Molecular Sciences

MDPI

Supplementary data

Hypoxia-Responsive Class III Peroxidases in Maize Roots: Soluble and Membrane-bound Isoenzymes

Anne Hofmann[1], Stefanie Wienkoop[2], Sönke Harder[3], Fabian Bartlog[1] and Sabine Lüthje[1,*]

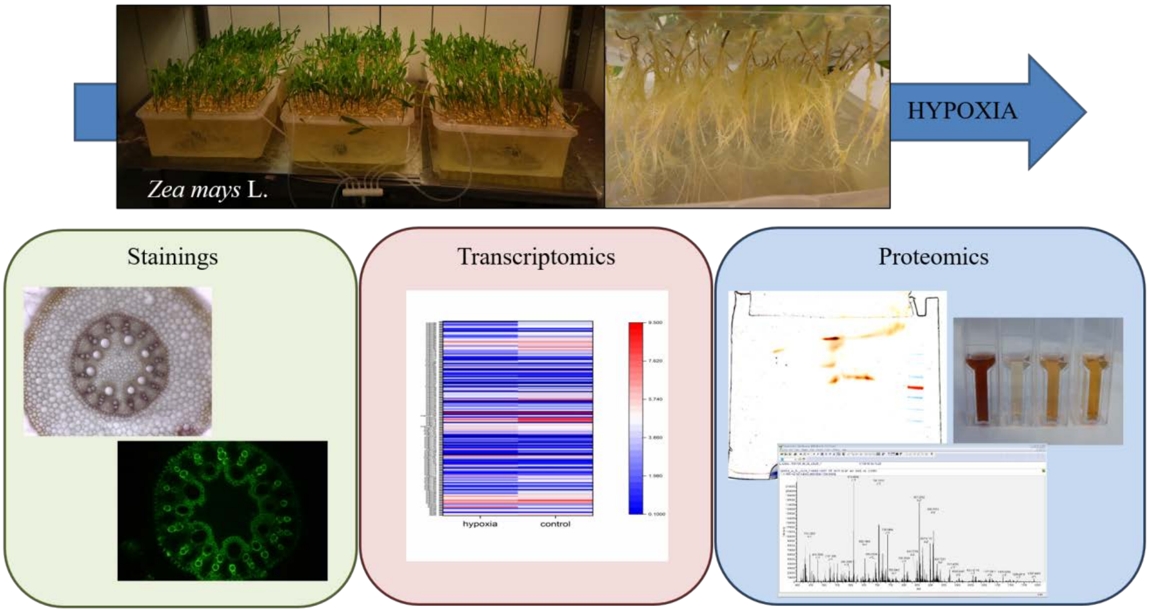

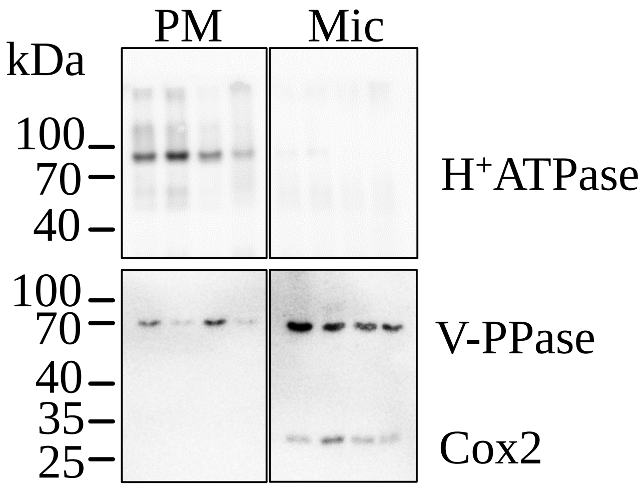

Figure S1 Western Blot. Washed plasma membranes and corresponding microsomes (5 µg) were incubated in 2x reducing loading buffer (32% glycerol, 4% SDS, 0.2 M Tris, 0.16% Bromphenol blue, 5% mercaptoethanol, pH 6.8) at room temperature for 30 min (H^+ATPase) or boiled at 95 ° C for 10 min (V-PPase, Cox2), separated on a 11% polyacrylamide gel (10 min 80 V and 90 min 120 V) and transferred to polyvinylidene difluoride membranes (Immobilon P^{SQ}, 0.2 µm pore size, Co. Merckmillipore, Darmstadt, Germany) using transfer buffer (25 mM Tris, 192 mM glycine, 0.01% SDS, 10% (v/v) methanol, pH 8.3) and semi-dry blotting system (Fastblot B33, Co. Biometra, An Analytik Jena Company, Germany) with 30 V and 150 mA for 1 h. Afterwards, membranes were blocked with 5% milk (instant skim milkpowder, Co. Frema, Herbolzheim, Germany) in TBST (50 mM Tris, 150 mM NaCl, 1% Tween 20, pH 7.6) at RT for 30 min and incubated with the first antibody in 5 mL TBST at 4 ° C over night.
Following antibodies were used to detect the plasma membane specific H^+ATPase of plants (H^+ATPase, Co. Agrisera, #AS07260, 1:5,000), the pyrophosphate-energized vacuolar membrane proton pump 1 (V-PPase, Co. Agrisera, #AS121849, 1:2,500) and the mitochondrial cytochrome c oxidase (Cox2, Co. Agrisera, #AS04053A, 1:1,000). Horse reddish peroxidase coupled goat anti rabbit served as secondary antibody (Co. Agrisera, Vännäs, Sweden, 1:25,000) prior to enhanced chemiluminescence (ECL) detection using ECL reagents (HRP-Juice, Co. PJK, Kleinblittersdorf, Germany) and the LAS-3000 Imaging system (Co. Fujifilm, Tokio, Japan). Shown are two biological replicates as representatives.

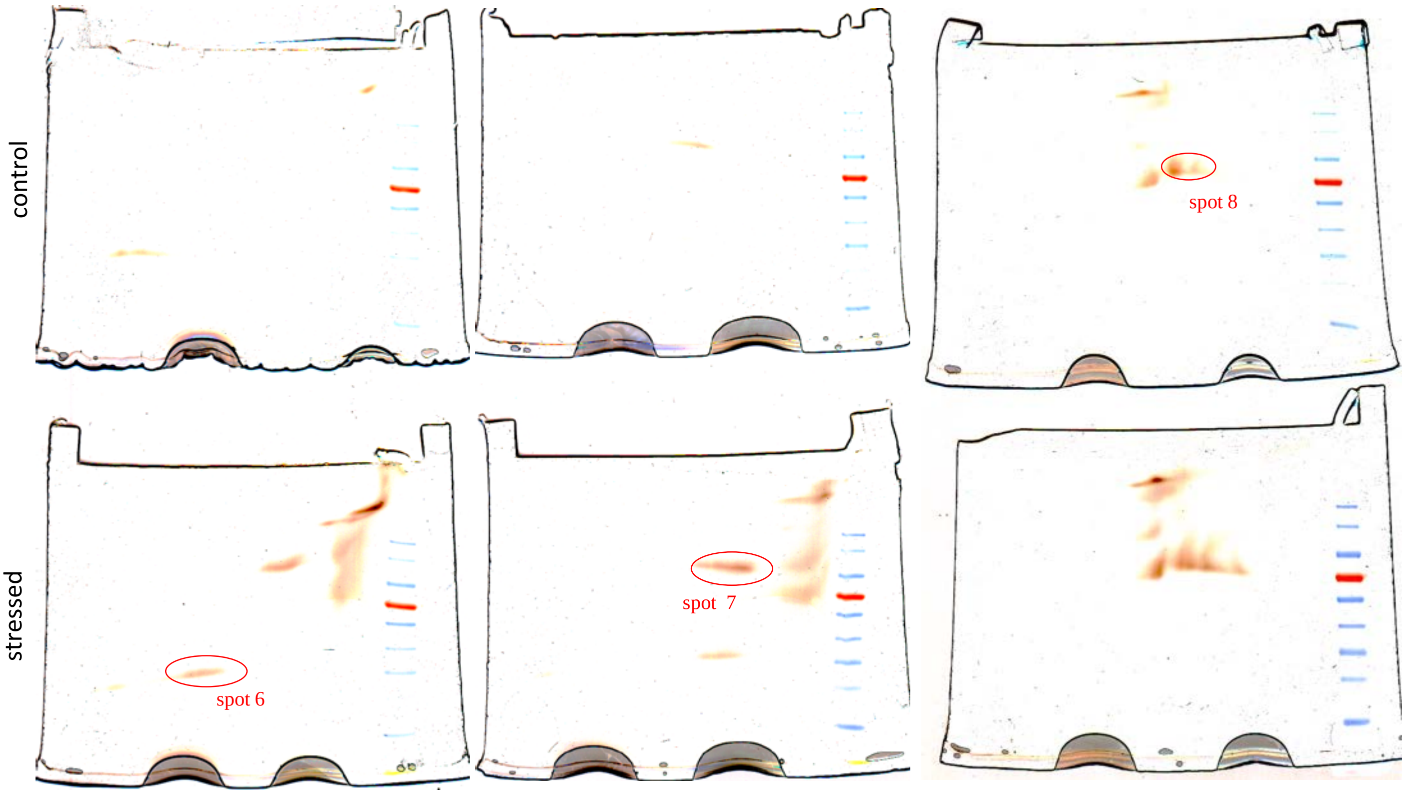

Figure S2 Abundance of guaiacol peroxidases under hypoxia-stress. Plasma membranes (250 µg total protein) of control and 24 h hypoxia-stressed maize roots were solubilised with 8% CHAPS for 1 h. The supernatant was separated on IEF gels pH 9-11 followed by 4-18% non-denaturating polyacrylamide gels in second dimensions. Class III peroxidases were visualized by staining with guaiacol. Besides five representative spots, that were found in all biological and technical replicates, some additional spots appeared in only some biological samples (spot 6-8). These were identified by mass spectrometry as follows: spot 6 with ZmPrx24 (B4FHG3), ZmPrx87 (B4FSW5), ZmPrx118 (B4FK72) and ZmPrx85 (A0A1D6E530), spot 7 with ZmPrx85 (A0A1D6E530) and spot 8 with ZmPrx01 (A5H8G4), respectively. CHAPS, 3-[(3-Cholamidopropyl)dimethylammonio]-1-propanesulfonate.

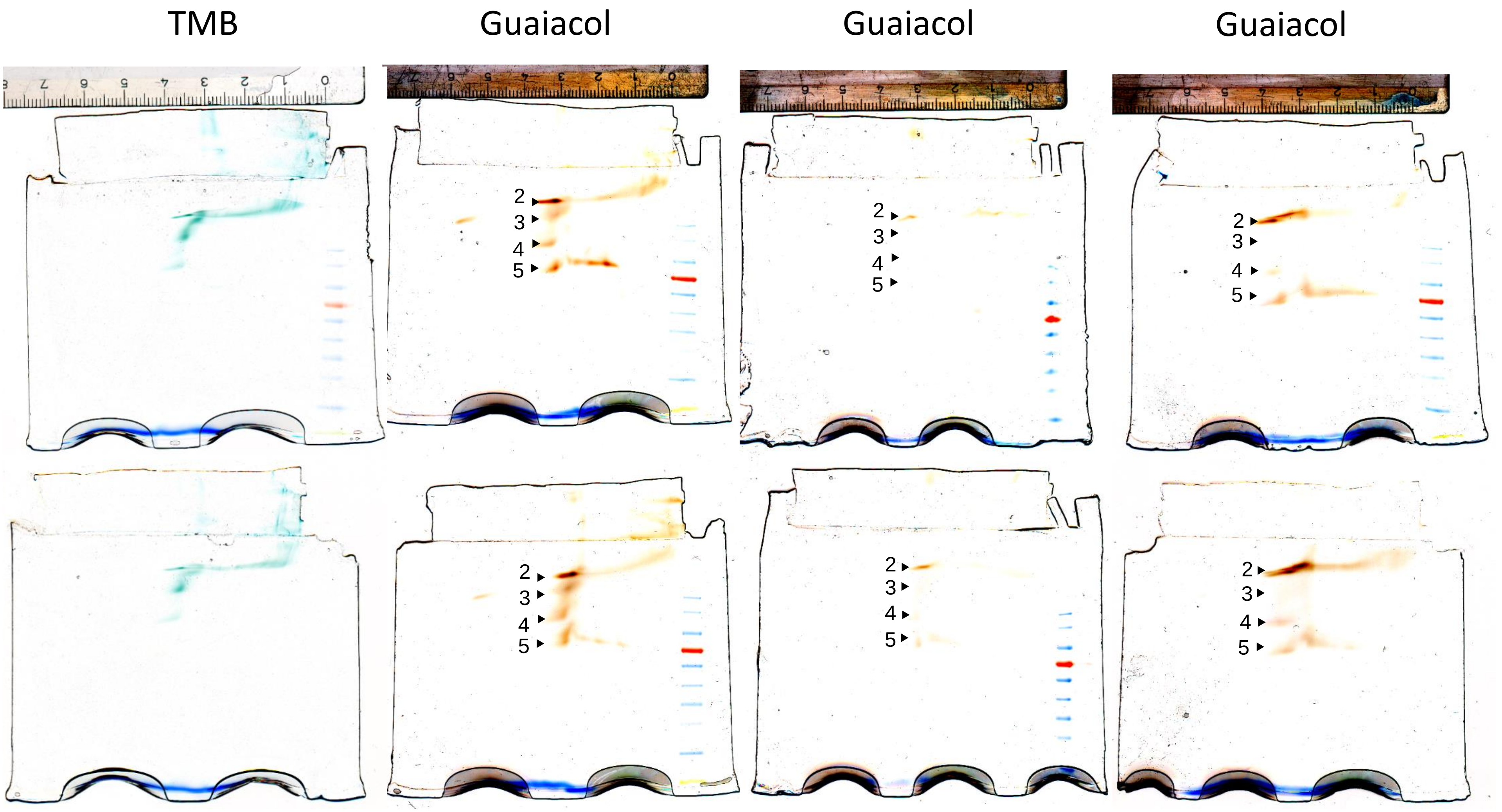

Supplement Figure 3A. Biological and technical replicates of 2D-PAGE pH 9-11. Plasma membrane (250 µg) of controls (upper panel) and 24 h hypoxia-stressed (lower panel) samples of biological replicate #1, solubilised with 8% CHAPS, separated on 2D-PAGE (isoelelctric focussing gel with pH from 9-11 followed by 4-18% non-denaturing gradient gel in second dimension) and stained with TMB for mass spectrometry analyses and guaiacol for class III peroxidases detection. Abundance of four spots (2-5) were analysed with ImageJ. CHAPS, 3-[(3-Cholamidopropyl)dimethylammonio]-1-propanesulfonate; TMB, 3,3′,5,5′-Tetramethylbenzidine.

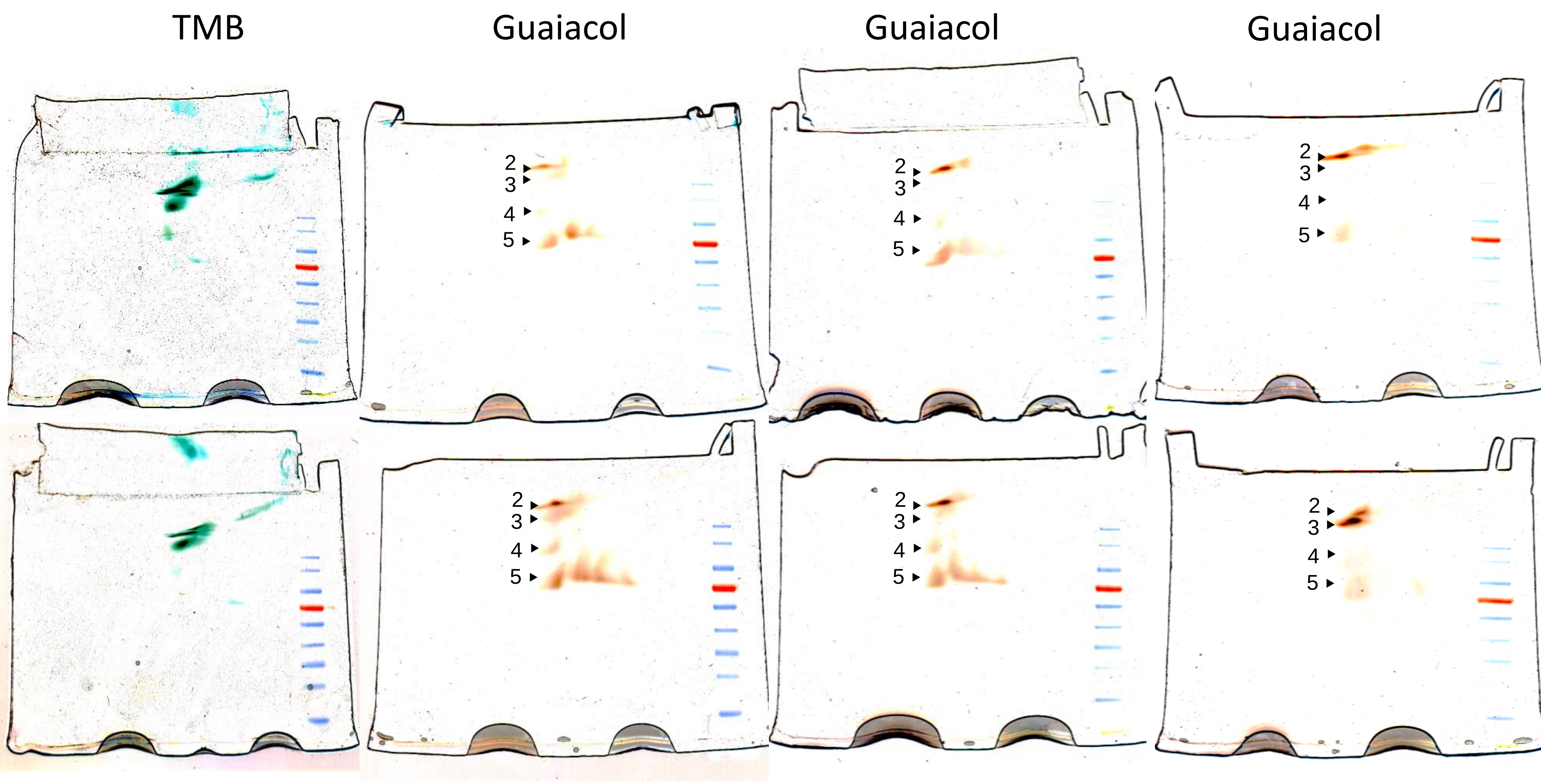

Supplement Figure 3B. Biological and technical replicates of 2D-PAGE pH 9-11. Plasma membrane (250 µg) of controls (upper panel) and 24 h hypoxia-stressed (lower panel) samples of biological replicate #2, solubilised with 8% CHAPS, separated on 2D-PAGE (isoelelctric focussing gel with pH from 9-11 followed by 4-18% non-denaturing gradient gel in second dimension) and stained with TMB for mass spectrometry analyses and guaiacol for class III peroxidases detection. Abundance of four spots (2-5) were analysed with ImageJ. CHAPS, 3-[(3-Cholamidopropyl)dimethylammonio]-1-propanesulfonate; TMB, 3,3′,5,5′-Tetramethylbenzidine.

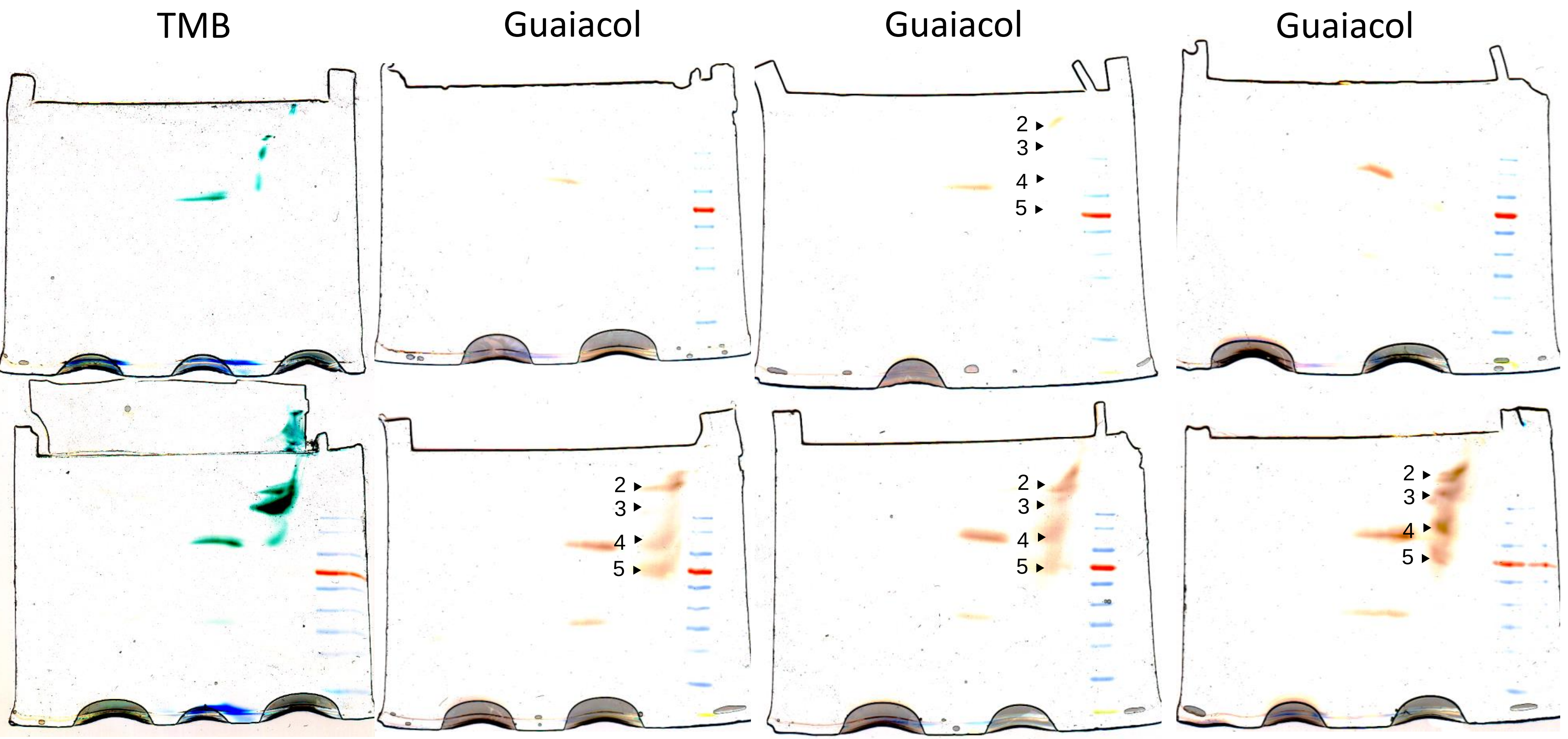

Supplement Figure 3C. Biological and technical replicates of 2D-PAGE pH 9-11. Plasma membrane (250 µg) of controls (upper panel) and 24 h hypoxia-stressed (lower panel) samples of biological replicate #3, solubilised with 8% CHAPS, separated on 2D-PAGE (isoelelctric focussing gel with pH from 9-11 followed by 4-18% non-denaturing gradient gel in second dimension) and stained with TMB for mass spectrometry analyses and guaiacol for class III peroxidases detection. Abundance of four spots (2-5) were analysed with ImageJ. CHAPS, 3-[(3-Cholamidopropyl)dimethylammonio]-1-propanesulfonate; TMB, 3,3′,5,5′-Tetramethylbenzidine.

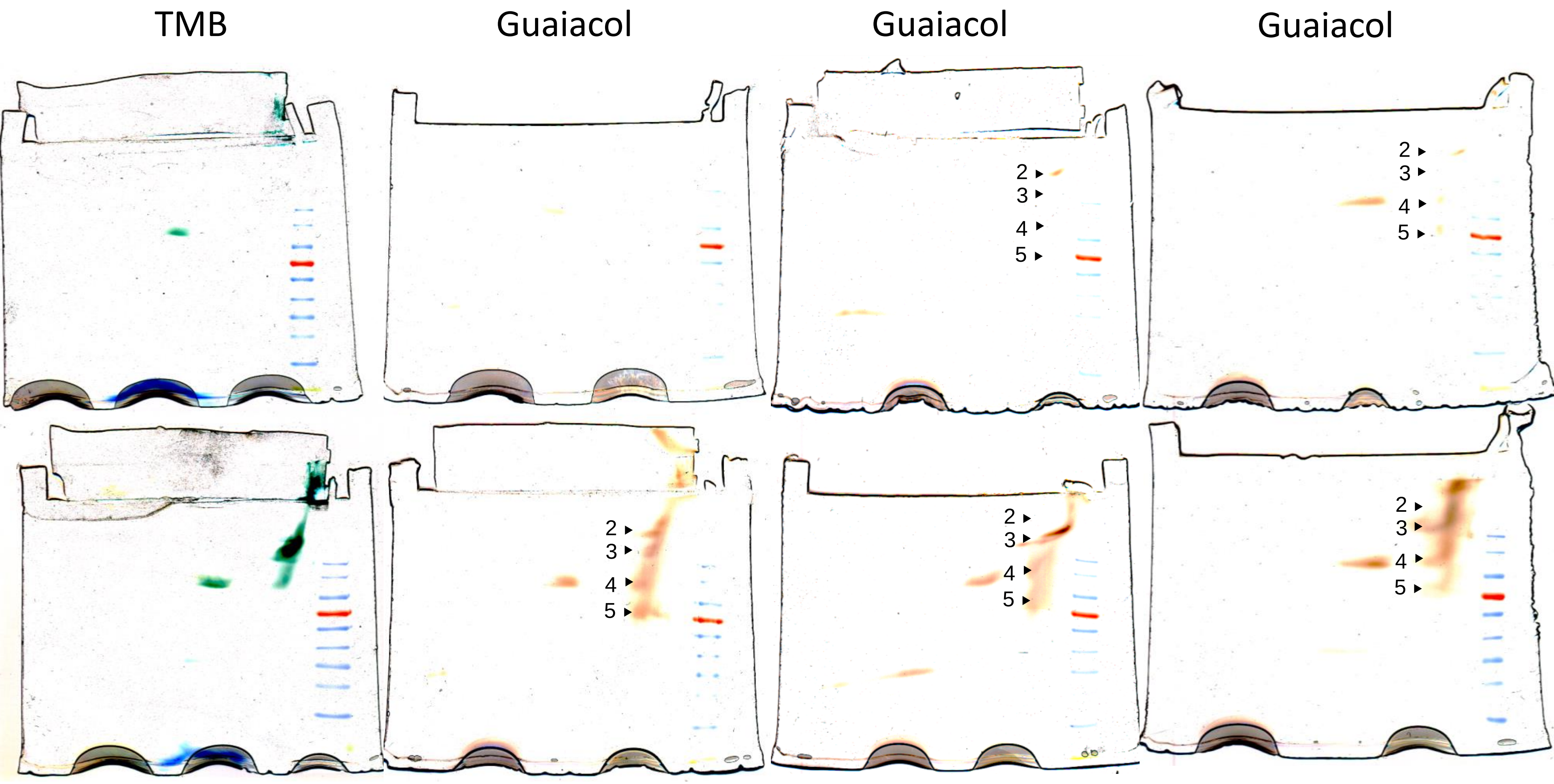

Supplement Figure 3D. Biological and technical replicates of 2D-PAGE pH 9-11. Plasma membrane (250 µg) of controls (upper panel) and 24 h hypoxia-stressed (lower panel) samples of biological replicate #4, solubilised with 8% CHAPS, separated on 2D-PAGE (isoelelctric focussing gel with pH from 9-11 followed by 4-18% non-denaturing gradient gel in second dimension) and stained with TMB for mass spectrometry analyses and guaiacol for class III peroxidases detection. Abundance of four spots (2-5) were analysed with ImageJ. CHAPS, 3-[(3-Cholamidopropyl)dimethylammonio]-1-propanesulfonate; TMB, 3,3′,5,5′-Tetramethylbenzidine.

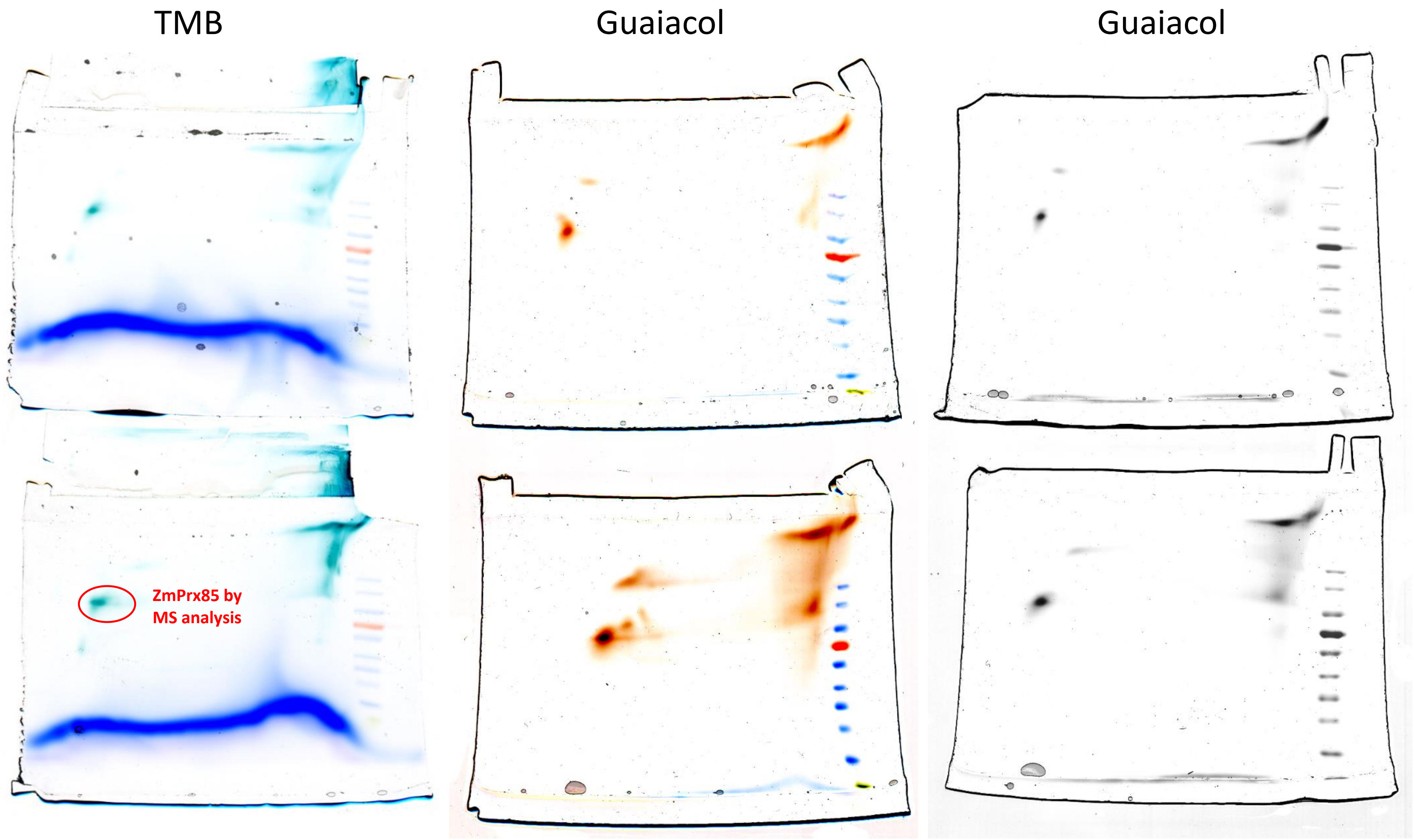

Supplement Figure 4A. Biological and technical replicates of 2D-PAGE pH 5-9. Plasma membrane (250 µg) of controls (upper panel) and 24 h hypoxia-stressed (lower panel) samples of biological replicate #1, solubilised with 8% CHAPS, separated on 2D-PAGE (isoelelctric focussing gel with pH from 5-9 followed by 4-18% non-denaturing gradient gel in second dimension) and stained with TMB for mass spectrometry analyses and guaiacol for class III peroxidases detection. Abundance of four spots (2-5) were analysed with ImageJ. CHAPS, 3-[(3-Cholamidopropyl)dimethylammonio]-1-propanesulfonate; TMB, 3,3′,5,5′-Tetramethylbenzidine.

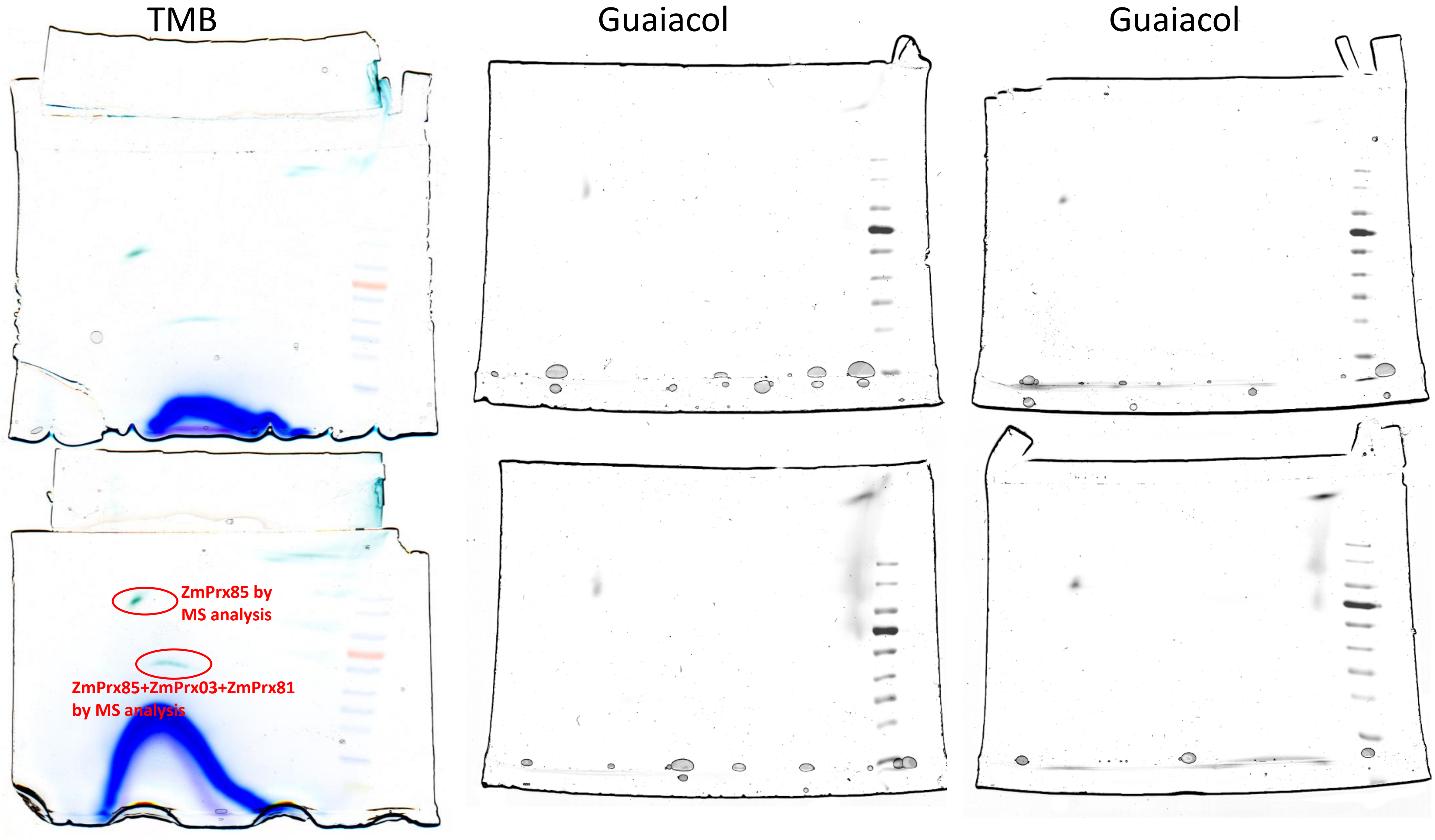

Supplement Figure 4B. Biological and technical replicates of 2D-PAGE pH 5-9. Plasma membrane (250 µg) of controls (upper panel) and 24 h hypoxia-stressed (lower panel) samples of biological replicate #2, solubilised with 8% CHAPS, separated on 2D-PAGE (isoelelctric focussing gel with pH from 5-9 followed by 4-18% non-denaturing gradient gel in second dimension) and stained with TMB for mass spectrometry analyses and guaiacol for class III peroxidases detection. Abundance of four spots (2-5) were analysed with ImageJ. CHAPS, 3-[(3-Cholamidopropyl)dimethylammonio]-1-propanesulfonate; TMB, 3,3′,5,5′-Tetramethylbenzidine.

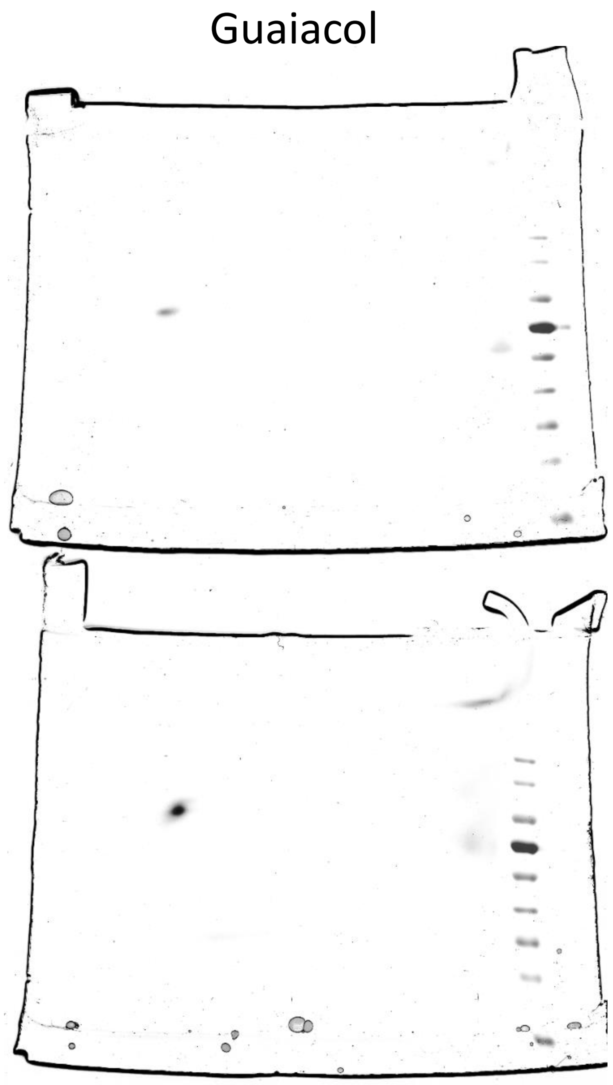

Supplement Figure 4C. Biological and technical replicates of 2D-PAGE pH 5-9. Plasma membrane (250 µg) of controls (upper panel) and 24 h hypoxia-stressed (lower panel) samples of biological replicate #3, solubilised with 8% CHAPS, separated on 2D-PAGE (isoelelctric focussing gel with pH from 5-9 followed by 4-18% non-denaturing gradient gel in second dimension) and stained with guaiacol for class III peroxidases detection. Abundance of four spots (2-5) were analysed with ImageJ. CHAPS, 3-[(3-Cholamidopropyl)dimethylammonio]-1-propanesulfonate.

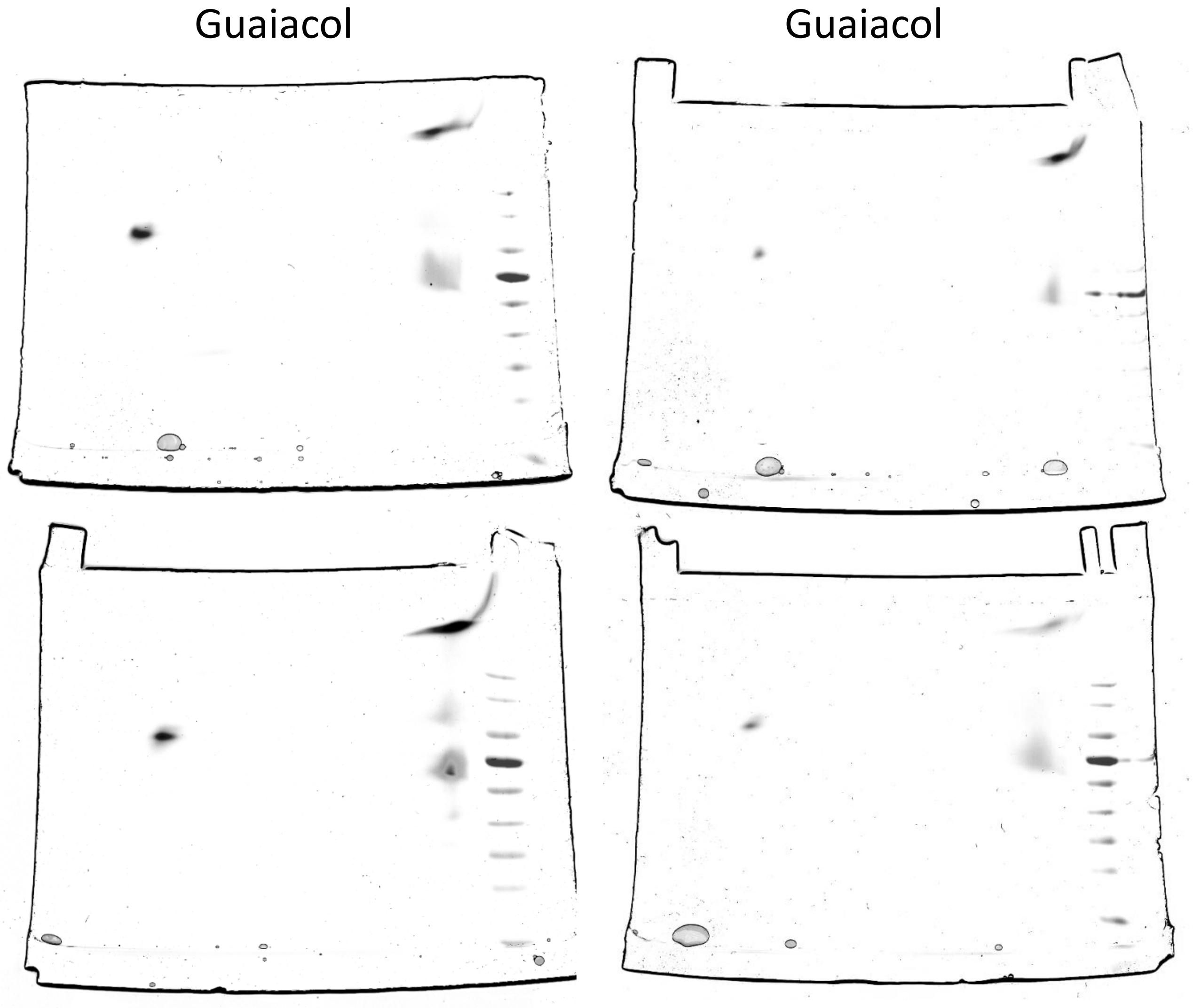

Supplement Figure 4D. Biological and technical replicates of 2D-PAGE pH 5-9. Plasma membrane (250 µg) of controls (upper panel) and 24 h hypoxia-stressed (lower panel) samples of biological replicate #4, solubilised with 8% CHAPS, separated on 2D-PAGE (isoelelctric focussing gel with pH from 5-9 followed by 4-18% non-denaturing gradient gel in second dimension) and stained with guaiacol for class III peroxidases detection. Abundance of four spots (2-5) were analysed with ImageJ. CHAPS, 3-[(3-Cholamidopropyl)dimethylammonio]-1-propanesulfonate.
